# IgGFc-binding protein and MUC2 mucin produced by colonic goblet-like cells spatially interact non-covalently and regulate wound healing

**DOI:** 10.3389/fimmu.2023.1211336

**Published:** 2023-06-08

**Authors:** Hayley Gorman, France Moreau, Antoine Dufour, Kris Chadee

**Affiliations:** ^1^ Department of Microbiology, Snyder Institute for Chronic Diseases, University of Calgary, Calgary, AB, Canada; ^2^ Department of Immunology and Infectious Diseases, Snyder Institute for Chronic Diseases, University of Calgary, Calgary, AB, Canada; ^3^ Physiology and Pharmacology, University of Calgary, Calgary, AB, Canada; ^4^ Biochemistry and Molecular Biology, University of Calgary, Calgary, AB, Canada

**Keywords:** MUC2 mucin, FCGBP, colitis, inflammation, wound healing

## Abstract

The colonic mucus bilayer is the first line of innate host defense that at the same time houses and nourishes the commensal microbiota. The major components of mucus secreted by goblet cells are MUC2 mucin and the mucus-associated protein, FCGBP (IgGFc-binding protein). In this study, we determine if FCGBP and MUC2 mucin were biosynthesized and interacted together to spatially enhance the structural integrity of secreted mucus and its role in epithelial barrier function. MUC2 and FCGBP were coordinately regulated temporally in goblet-like cells and in response to a mucus secretagogue but not in CRISPR-Cas9 gene-edited *MUC2 KO* cells. Whereas ~85% of MUC2 was colocalized with FCGBP in mucin granules, ~50% of FCGBP was diffusely distributed in the cytoplasm of goblet-like cells. STRING-db v11 analysis of the mucin granule proteome revealed no protein-protein interaction between MUC2 and FCGBP. However, FCGBP interacted with other mucus-associated proteins. FCGBP and MUC2 interacted via N-linked glycans and were non-covalently bound in secreted mucus with cleaved low molecular weight FCGBP fragments. In *MUC2 KO*, cytoplasmic FCGBP was significantly increased and diffusely distributed in wounded cells that healed by enhanced proliferation and migration within 2 days, whereas, in WT cells, MUC2 and FCGBP were highly polarized at the wound margin which impeded wound closure by 6 days. In DSS colitis, restitution and healed lesions in *Muc2^+/+^* but not *Muc2^-/-^* littermates, were accompanied by a rapid increase in *Fcgbp* mRNA and delayed protein expression at 12- and 15-days post DSS, implicating a potential novel endogenous protective role for FCGBP in wound healing to maintain epithelial barrier function.

## 1 Introduction

The gastrointestinal tract digests and absorbs nutrients critical to basic functioning and survival. Adding to the complexity of this task, the gut is open to the external environment, requiring constant separation from noxious agents and potentially harmful microorganisms from the single layer of mucosal epithelial cells. Covering the epithelial cells in the intestine is MUC2 mucus secreted by goblet-like cells that contains a variety of mucus-associated proteins (MAPs), antimicrobial peptides, and host microbiota providing separation and is therefore vital in host protection and innate immunity. The MUC2 mucus interface is critical for maintaining host health and homeostasis by not only providing a protective shield for epithelial cells but also serving as a food source and substrate for indigenous microbiota (1–3). Aptly, disruption of the mucus layer is often associated with infectious colitis, inflammatory bowel diseases, and metabolic disorders (1, 4–6).

MUC2 is a large complex glycoprotein [Mr > 10 (6)] with carbohydrates contributing up to 80% of its molecular weight. MUC2 is packaged and stored in mucin granules of goblet-like cells prior to its release into 2 layers in the colon: an inner dense stratified sterile layer attached to epithelial cells and an outer loose layer that houses the microbiota (7). The MUC2 peptide core is synthesized in the rough endoplasmic reticulum where mucin monomers rapidly form dimers following N-linked glycosylation. O-linked sugars and sialic acid are added after the dimers are transferred to the Golgi apparatus. Several oligosaccharide chains are attached at their non-reducing ends to the peptide backbone by the first sugar residue GalNAc via an *O*-glycosidic linkage to serine and threonine residues (8, 9). In addition to glycans, the MUC2 apoprotein is composed of 2 mucin domains. Each domain contains the variable number tandem-repeat region (VNTR) and the irregular repeat (IR) region that is composed of proline, threonine, and serine residues (PTS) that become heavily glycosylated (3). Unlike the protein core that is heavily O-glycosylated, the N- and C-terminal regions are composed mainly of cysteine-rich domains and N-linked glycans that are referred to as von Willebrand D domains. The cysteine-rich areas are critical for allowing intermolecular disulfide bridges to form between MUC2 monomers, forming dimers, and subsequently, MUC2 polymers that are resistant to trypsin and other digestive enzymes and gives the MUC2 mucin network stability and its viscoelastic properties (10). More recently, it is becoming clear that goblet cells also produce a variety of other proteins that are present in the mucus layer but their functions are not well known. These MAPs include calcium-activated chloride channel regulator 1 (CLCA1), kallikrein 1 (KLK1), zymogen granule 16 (ZG16), anterior gradient 2 (AGR2), and F_c_ fragment of IgG binding protein (FCGBP) (5, 11). Of these proteins, FCGBP is of particular interest due to its similar structure to MUC2 (12), but its role in colonic mucus and function remains elusive. FCGBP is reportedly around 300 kDa and similar to MUC2, it contains multiple von Willebrand factors and mucin-like repeats (CGLCGN) (12). MUC2 contains a Gly-Asp-Pro-His (GDPH) amino acid sequence in its von Willebrand domain within the C-terminus that is autocatalytically cleaved (13, 14). Recent studies have identified that human FCGBP has a GDPH sequence in 11 out of 13 of its von Willebrand domains that undergo autocatalytic cleavage, similar to MUC2 (12, 15). The cleaved sequences are secreted and stabilized via disulfide bonds (16). Additionally, it has 33 potential N- but no O-linked glycosylation sites. The abundance of N-glycosylation sites in FCGBP suggests it could interact with MUC2 N-linked glycans to form covalent and/or non-covalent bonds, strengthening the mucus layer. However, it is still unclear if FCGBP biochemically interacts with MUC2 or what functional role it plays in the cell or the mucus layer.

A major knowledge gap in colonic mucus biology is whether MAPs are goblet cell-secreted proteins that become intermixed innocuously with secreted mucus or whether they are structurally associated with MUC2. It is unclear if MAPs are present and packaged in MUC2 mucin granules. Based on this deficiency and the strong similarity of FCGBP to MUC2 mucin, we hypothesized that FCGBP plays an integral part alongside MUC2 mucin to maintain the structural integrity of the colonic mucus gel. Here we reveal by biochemical and proteomic analysis that FCGBP and MUC2 are coordinately biosynthesized, non-covalently bound, packaged within MUC2 mucin granules, and are secreted together to form part of the structural component of the mucus layer. In wound healing assays and an animal model of colitis, cytoplasmic FCGBP not bound to MUC2 played an endogenous role in wound healing to maintain epithelial barrier function.

## 2 Materials and methods

### 2.1 Ethics statement and animals

In all experiments, 10 to 12-week-old C57BL/6 *Muc2^+/+^* and *Muc2^-/-^* littermates (equal numbers of male and female mice) were used. Wild-type mice were purchased from Charles River Laboratories International (St-Constant, Quebec, Canada) and *Muc2^-/-^* mice obtained from Dr. Velcich, were bred in-house to generate littermate *Muc2^+/-^* breeding pairs and backcrossed on a C57BL/6 background. Germ-free (GF) mice on a C57BL/6 background were purchased from the International Microbiome Centre at the University of Calgary. Mice were kept in sterilized, filter-top cages and maintained on standard food and water *ad libitum* in specific-pathogen-free (SPF) conditions. The Health Sciences Animal Care Committee from the University of Calgary examined the animal care and treatment protocol (AC18-0218) and approved the experimental procedures proposed and certifies with the applicant that the care and treatment of animals used were in accordance with the principles outlined in the most recent policies on the “Guide to the Care and Use of Experimental Animals” by The Canadian Council on Animal Care.

### 2.2 Human goblet-like cells

The human colon cancer cell line LS174T was maintained in Eagle’s Minimum Essential Medium (EMEM) supplemented with 10% heat-inactivated fetal bovine serum, 10 mM HEPES, and 10 mM penicillin and streptomycin (complete EMEM). Cells were maintained in a humidified incubator with 5% CO_2_. Media was changed every 2 to 3 days and cells were passed once a week, as the cells became confluent. For protein extraction, LS174T cells were plated onto 6-well plates (2.5 x 10^5^ cells/well) in 3 mL of complete EMEM. For RNA extraction, LS174T cells were plated onto 12-well plates (1.5 x 10^5^ cells/well) in 2 mL of complete EMEM. 10 μM PMA was added to wells for 2 hours to induce MUC2 expression where indicated. Tunicamycin (5 μg/mL) was added to wells for 12 hours to interrupt *N-*glycosylation where indicated.

### 2.3 Quantitative real-time PCR

RNA from cells were extracted using the Omega Bio-Tek E.Z.N.A. Total RNA Kit, as per the manufacturer’s instructions. RNA from tissues were homogenized in Ribozol RNA Extraction Reagent (VWR) and then extracted as per the manufacturer’s instructions. For DSS-treated tissue, the DSS was removed as previously described (17). Briefly, RNA was incubated with 2 M lithium chloride on ice for 2 hours, followed by centrifugation at 14 000 x *g* for 30 min at 4 °C. The supernatant was removed and discarded, and the pellet of RNA was dissolved in 200 μL RNase-free water. The purification process was repeated once more. The pelleted RNA was resuspended in 200 μL RNase-free water and precipitated at -20°C for 30 min in 3 M sodium acetate and 100% ethanol. Once the RNA was isolated, RNA yield was measured using a NanoDrop1000 spectrophotometer. 500 ng of RNA was reverse transcribed using the qScript cDNA SuperMix (QuantaBio). qPCR was performed using primers the following primers and annealing temperatures: Human MUC2 (F: CAGCACCGATTGCTGAGTTG, R: GCTGGTCATCTCAATGGCAG, 56°), Human FCGBP (F: GGACCTCAAGAACACTGGCA, R: GAGGATGGAGACTGAAGCGG, 60°), Human GAPDH (F: TGATGACATCAAGAAGGTGGT-GAAG, R: TCCTTGGAGGCCATGTGGGCCAT, 60°), Human MATH1 (F: TGCACTTCTCGAC-TTTCGAGGACA, R: AACTTGCCTCATCCGAGTCACTGT, 63°), Human SPDEF (F: TTGCTA-CTCAAGCCCCACAG, R: CCGGATGATGCCCTTCTTGT, 58°), Human ATF4 (F: TGCTGTCT-GCCGGTTTAAGT, R: CTGCTGCCTCTAATACGCCA, Mouse Muc2 (F: GAAGCCAGATCCCG-AAACCA, R: CCAGCTTGTGGGTGAGGTAG, 60°), Mouse Fcgbp (F: GACAAAGCCTATCT-CTGCCGT, R: CTTGGGTAGCAGACAGGGAA, 58°), Mouse β-Actin (F: TGGGGTGTTGAAGG-TCTC, R: CTACAATGACTGCGTGTG, 58°). Mouse Math1 (F: AAAGGAGGCTGGCAG-CAA, R: TGGTTCAGCCCGTGCAT, 58°), Mouse Spdef (F: GACTCACACTCAAGGGGCAA, R: TCAG-AAGAGTCGTCCGTCCT, 58°). Reactions were done in a Corbett Rotor Gene 3000 system. Data analysis was done using the 2^−ΔΔCT^ method to interrogate fold changes in gene expression.

### 2.4 Western blots

Protein samples were taken from 6-well plates after various times and treatments. The lysates were extracted, washed with cold PBS, and subsequently lysed with 300 μL complete lysis buffer (1 mmol/L EDTA, 20 mmol/L Tris-HCl, 0.1% SDS, 100 mmol/L NaCl, 0.5% Triton X-100, phenylmethylsulfonyl fluoride, and a protease inhibitor cocktail; Sigma-Aldrich) on ice for 30 min. Samples were then centrifuged at 8000 x *g* for 10 min and the pelleted debris was removed. The remaining protein was quantified by a BCA protein assay and adjusted to equal concentrations of protein, and then resuspended into 5X Laemmli buffer and boiled for 5 min. The lysates were loaded into 7.5%, 10%, 12%, or 15% SDS polyacrylamide gels and then transferred onto a nitrocellulose membrane. After transfer, the membranes were then blocked in 5% milk powder in PBS and 0.1% Tween. The membranes were then probed with primary antibodies: MUC2 (in-house antibody against CsCl purified glycosylated MUC2 mucin (18) or Santa Cruz sc-515106 for the C-terminus), FCGBP (Cloud-Clone PAP389Hu01), and CLCA1 (Abcam 1808521). As a housekeeping marker, mouse monoclonal anti-glyceraldehyde 3-phosphate dehydrogenase (GAPDH) (Calbiochem 6C5) was used. Corresponding HRP-conjugated secondary antibodies were diluted in 3% milk powder in PBS and 0.1% Tween and then visualized using ChemiLucent ECL detection (EMD Millipore) and imaged on a Bio-Rad ChemiDoc Imager. For samples prepared under non-reducing conditions, β-Mercaptoethanol and SDS were omitted from the 5X sample buffer.

### 2.5 Confocal and super-resolution microscopy

1.5x10^5^ cells were seeded on sterile No. 1.5 glass coverslips and left to grow for 24 hours. Coverslips were washed twice with ice-cold PBS, then fixed with 4% paraformaldehyde for 15 min at 37°C, and then washed twice with PBS again. Cells were then permeabilized with PBS containing 0.35% Triton for 5 min at room temperature. After washing thrice with PBST (PBS with 0.1% Tween), coverslips were blocked for 1 hour with 5% donkey serum in PBST, washed twice, and incubated overnight with primary antibodies for MUC2 (sc-515106, Santa Cruz) or FCGBP (PAP389Hu01, Cloud-Clone) in a humidified chamber at 4°C. The next day, coverslips were washed with PBST thrice and incubated for 1 hour with fluorescent secondary antibodies and DAPI (Life Technologies). For migration and proliferation studies, cells were counterstained with Paxillin (05-417, Millipore) and the incorporation of EdU quantified as per the manufacturer’s instruction (C10637, Thermo Fisher), respectively. Coverslips were mounted with FluorSave (Calbriochem) and imaged using a Nikon A1R confocal laser scanning microscope or Nikon Ti Eclipse Widefield microscope. STED imaging used the same protocol; however, imaging was performed using an Aberrior STEDYCON microscope attached to a Nikon Ti Eclipse Widefield microscope. Quantification and image analysis were done in FIJI (ImageJ) to determine protein intensity and colocalization.

### 2.6 Preparation of mucin granules

Mucin granules were prepared from confluent flasks of LS174T cells. Cells were washed with PBS, collected, and centrifuged at 300 x g for 5 min. The resulting pellet was resuspended in MES homogenization buffer (250 mM sucrose, 25 mM MES, 0.1 mM MgSO4, 2 mM EGTA, 0.1 mM PMSF) and homogenized in a Dounce homogenizer. The homogenate was then centrifuged at 300xg for 5 min and the supernatant was collected and re-centrifuged at 300xg for 10 min. The cleared supernatant was then centrifuged at 2100 x g for 15 min. The resulting pellet was then washed 3 times with PBS and the crude mucin granules were either left untreated or treated with either 1% CHAPS or 1% octyl-glucoside. For further purification, the crude granules were resuspended into 2 mL HEPES buffer (20 mM HEPES, 137 mM NaCl) and carefully placed on top of a sucrose gradient (2 mL fractions of each 2 M sucrose, 1.5 M sucrose, 1 M sucrose, and 0.5 M sucrose) and ultracentrifuge at 20 000xg overnight. After centrifugation, 1 mL fractions were isolated, washed three times in PBS, and prepared for analysis.

### 2.7 Shotgun proteomics analysis

WT and *MUC2 KO* LS174T goblet-like cells were used for shotgun proteomics analysis. Protein samples were lysed with 1% SDS, 0.1 M EDTA in 200 nM HEPES (pH 8), and protease inhibitor tablets (Roche). Proteins were denatured with the addition of a final concentration of 10 mM DTT. Samples were alkylated by incubation with a final concentration of 15 mM iodoacetamide in the dark for 25 min at room temperature at a pH of 6.5. Next, to label peptide α- and ϵ-amines, samples were incubated for 18 h at 37°C with isotopically heavy [40 mM 13CD2O + 20 mM NaBH3CN (sodium cyanoborohydride)] or light labels [40 mM light formaldehyde (CH2O) + 20 mM NaBH3CN]. These were all final concentrations. Samples were subjected to C18 chromatography before being subjected to liquid chromatography and tandem mass spectrometry (LC-MS/MS).

### 2.8 Mass spectrometry

All MS experiments were carried out by the Southern Alberta Mass Spectrometry (SAMS) core facility at the University of Calgary, Canada as previously described (19). Analysis was performed on an Orbitrap Fusion Lumos Tribrid MS (Thermo Scientific) operated with Xcalibur (version 4.0.21.10) and coupled to a Thermo system. Tryptic peptides (2 μg) were loaded onto a C18 trap (75 μm x 2 cm; Acclaim PepMap 100, P/N 164946; ThermoScientific) at a flow rate of 2 μl/min of solvent A (0.1% formic acid and 3% acetonitrile in LC-MS grade water). Peptides were eluted using a 120 min gradient from 5 to 40% (5–28% in 105 min followed by an increase to 40% B in 15 min) of solvent B (0.1% formic acid in 80% LC-MS grade acetonitrile) at a flow rate of 0.3 μL/min and separated on a C18 analytical column (75 μm x 50 cm; PepMapRSLC C18; P/N ES803; Thermo Scientific). Peptides were then electrosprayed using 2.3 kV voltage into the ion transfer tube (300 °C) of the Orbitrap Lumos operating in positive mode. The Orbitrap first performed a full MS scan at a resolution of 120,000 FWHM to detect precursor ions with an m/z between 375 and 1575 and a +2 to +7 charge. The Orbitrap AGC (Auto Gain Control) and the maximum injection time were set at 4 x 105 and 50 ms, respectively. The Orbitrap was operated using the top speed mode with a 3 s cycle time for precursor selection. The most intense precursor ions presenting a peptidic isotopic profile and having an intensity threshold of at least 5000 were isolated using the quadrupole and fragmented with HCD (30% collision energy) in the ion routing multipole. The fragment ions (MS2) were analyzed in the ion trap at a rapid scan rate. The AGC and the maximum injection time were set at 1 x 104 and 35 ms, respectively, for the ion trap. Dynamic exclusion was enabled for 45 s to avoid the acquisition of the same precursor ion with a similar m/z (plus or minus 10 ppm).

### 2.9 Proteomic data and bioinformatics analysis

Spectral data were matched to peptide sequences in the human UniProt protein database using the MASCOT (www.matrixscience.com). The cleavage site specificity was set to Trypsin/P for the proteomics data, with up to two missed cleavages allowed. After protein identification, the resulting hits were submitted to Metascape (20) for bioinformatic analysis to generate the gene ontology pathway analyses and transcription factor analysis presented. For the protein-protein interaction study, STRING v11 (https://string-db.org) software was used (21).

### 2.10 Preparation of native LS174T mucins

Mucin preparations from LS174T secretions were collected as previously described (22). Briefly, spent media from LS174T cells were dialyzed, lyophilized, resuspended in column buffer, and applied to a Sepharose 4B columns (50 by 2.5 cm; Bio-Rad Laboratories) that were equilibrated with column buffer at 4°C. The column was calibrated by using known standards: blue dextran (2000 kDA), thyroglobulin (669 kDa), bovine serum albumin (67 kDa), and Chymotrysinogen (25 kDa). Four mL fractions were collected and read on a DU800 Spectrophotometer (Beckman Coulter). Appropriate samples were pooled and dialyzed against deionized water at 4°C overnight, lyophilized, and resuspended into 100 µL of water. Protein concentration was determined through a BCA protein assay prior to preparing samples for Western blotting.

### 2.11 Organoid growth and cultivation

Colonic organoids were derived as previously described from *Muc2^+/+^* and *Muc2^-/-^* littermates (23, 24). Briefly, the colons were excised, and crypts digested using a gentle cell dissociation buffer (StemCell Technologies) and approximately 100 crypts were embedded into GFR-matrigel (BD) domes. Organoids were fed every two days with advanced DMEM supplemented with L-WRN (ATCC CRL-3276) as well as N2, B27, GlutaMax, SB202190, Nicotinamide, N-acetylcysteine, A83-01, and mEGF. Colonoids were passaged once every 7-10 days with TrypeLE. For experiments, colonoids were grown for 7 days in complete media and then fed with L-WRN-conditioned media containing only N2, B27, Nicotinamide, and Glutamax for 3 days. Five µM DAPT was administered to the experimental media 24 hours prior to harvesting for experiments using colonoids skewed to a goblet cell phenotype.

### 2.12 Luminex analysis of growth factors

Production and measurement of growth factors by WT and MUC2 KO LS174T cells were assessed using a Luminex multiplex assay (Eve Technologies, Calgary, AB, Canada). Wounds were generated in cells using a 2-well silicone insert with a defined 500 µm cell-free gap (IBIDI), and after 48 hours, the culture inserts were removed, and the supernatants were collected for day 0 measurements. Fresh media were added and removed on days 2 and 4. The supernatant was centrifuged at 10 000 x *g* for 10 min to remove cellular debris prior to the analysis.

### 2.13 Antibiotics treatment

The animals were treated with an antibiotic cocktail as previously described to decrease the bacterial load (25). Briefly, the mice were gavaged every 12 hours with an antibiotic cocktail. For the first 3 days, the cocktail contained amphotericin-B (1 mg/kg). Starting on day 4, the cocktail contained vancomycin (50 mg/kg), neomycin (100mg/kg), metronidazole (100 mg/kg), and amphotericin-B (1 mg/kg) for the next 14 days. Additionally, ampicillin (1 mg/mL) was added to the drinking water and consumed *ad libitum*. The animals were used 2 days following the antibiotic treatment in order for it to be cleared from systemic circulation.

### 2.14 Induction of colitis

Chemical colitis was induced as previously described (26) using DSS (MW: 36,000–50,000; MP Biochemicals, Santa Ana, CA) dissolved in tap water. The *Muc2^+/+^* animals received 3.5% for 5 days, while the *Muc2^-/-^* mice received 1% for 3 days follow up by water ad libitum. The mice were weighed daily and sacrificed on days 4, 8, 12, and 15 post-DSS.

### 2.15 Statistical analysis

All experiments were repeated at least 3 independent times. All statistical analysis was done using GraphPad Prism 8. Statistical significance between two or more groups was analyzed with two-way ANOVA, whereas the Student’s t-test was used for comparison between 2 groups. Significance was assumed at P < 0.05. Error bars depict mean ± standard deviation (SD).

## 3 Results

### 3.1 MUC2 and FCGBP expression are coordinately regulated in goblet-like cells

**Figure 1:**
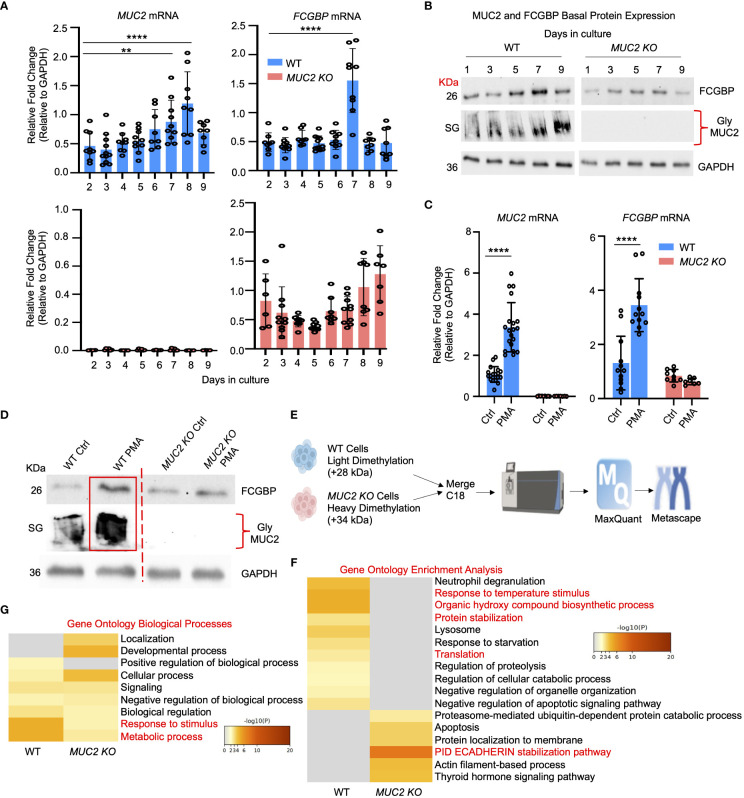
MUC2 and FCGBP expression are coordinately regulated *in vitro*. **(A)** Temporal mRNA expression of MUC2 and FCGBP in WT and CRISPR-Cas9 gene-edited *MUC2 KO* human LS174T goblet-like cells in culture. mRNA was isolated from cells daily for 9 days (n=8). **p < 0.01, ****p < 0.0001. **(B)** Temporal MUC2 and FCGBP protein expression in LS174T goblet-like cells in culture. Protein was quantified every 2 days. GAPDH was used as a loading control. SG = stacking gel. Gly MUC2 = glycosylated MUC2 antibody (n=5). **(C)** mRNA expression of MUC2 and FCGBP in LS174T goblet-like cells basally and in response to PMA. qPCR was performed to quantify the most abundant mRNA transcripts for MUC2 and FCGBP and both were upregulated in response to the mucus secretagogue PMA in WT cells (n=10). ****p < 0.0001. **(D)** PMA upregulated protein expression of both MUC2 and FCGBP in LS174T goblet-like cells. Cell lysates were analyzed by SDS-PAGE/Western blot with antibodies against FCGBP and MUC2. GAPDH was used as a loading control. SG = stacking gel (n=5). **(E)** Workflow for proteomics analysis to compare WT and *MUC2 KO* cell lysates. WT cells were tagged with a light dimethylation tag, and *MUC2 KO* cells were tagged with a heavy dimethylation tag. Both cells were run through the mass spectrometer, and the results were run through MaxQuant and Metascape. **(F)** Metascape analysis showing gene ontology enrichment analysis associated with WT and *MUC2 KO* cells. **(G)** Metascape analysis showing gene ontology biological processes utilized by WT and *MUC2 KO* cells. Pathways of interest are highlighted in red.

**Figure 2:**
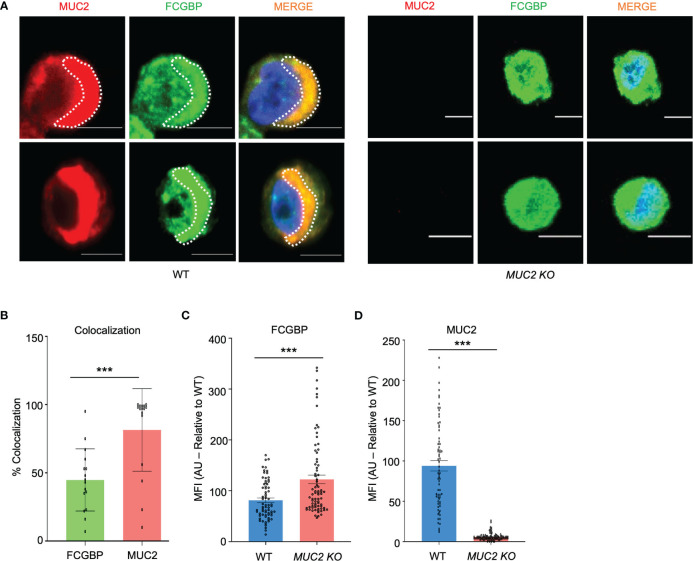
MUC2 and FCGBP localization within goblet-like cells. **(A)** Confocal microscopy images of WT and *MUC2 KO* LS174T cells showing MUC2 (red), FCGBP (green), and DAPI (blue). Note FCGBP bound to MUC2 and free FCGBP in the cytoplasm of goblet-like cells. Images are representative of 5 different experiments. Scale bar = 10 μm. **(B)** Quantification of colocalization between MUC2 and FCGBP in WT cells by confocal microscopy images. ***p<0.001 (n=15). **(C)** Mean fluorescence intensity (MFI) of FCGBP and **(D)** MUC2 compared to WT cells of confocal images taken in **(A)** ***p<0.001 (n=15).

Even though FCGBP has been referred to as a mucus-associated protein, no studies to date have reported if *FCGBP* and *MUC2* mRNA expression are coordinately regulated or if the proteins are secreted together in colonic mucus constitutively, and in response to a mucus secretagogue. To address this possibility, we quantified the temporal expression of *MUC2* and *FCGBP* mRNA in WT (27) and CRISPR/Cas9 *MUC2 KO* LS174T goblet-like cells (*MUC2 KO*) in culture (23). Following a lag phase from 2 to 6 days, *MUC2* and *FCGBP* mRNA expression in WT cells significantly peaked on days 8 and 7, respectively (**Figure 1A**). In contrast, *FCGBP* mRNA expression in *MUC2 KO* cells showed no significant changes in expression between days 2 and 9. To determine if *MUC2* and *FCGBP* mRNA expression correlated with protein production, Western blots were done every 2 days that revealed FCGBP and glycosylated MUC2 (Gly MUC2) protein expression in WT cells increased temporally with peak expression at day 7 consistent with mRNA expression (**Figures 1A, B**, **Supplementary Figure 1A**). In *MUC2 KO* cells, FCGBP protein expression similarly increased temporally with peak expression at day 7 (**Figure 1B**). To determine if *FCGBP* gene expression was inducible similar to *MUC2* (28), cells were stimulated with the MUC2 secretagogue, phorbol 12-myristate 13-acetate (PMA) (29). Surprisingly, PMA significantly upregulated the expression of both *MUC2* and *FCGBP* mRNA and protein production in WT goblet-like cells, whereas *FCGBP* mRNA expression and protein were unchanged in *MUC2 KO* cells (**Figures 1C, D**, **Supplementary Figure 1B**). These results demonstrate that both MUC2 and FCGBP associate with each other in WT goblet-like cells and are secreted together in response to a mucus secretagogue. This was in marked contrast to what was observed in *MUC2 KO* cells. To determine the proteins that were basally expressed in WT and *MUC2 KO* cells, the global proteome was quantified by quantitative shotgun proteomic analysis (**Figure 1E**, **Supplementary Table 1**). WT cells were metabolically active and among the most abundant proteins that were upregulated were MUC2 mucin and heat shock protein beta-1, whereas in *MUC2 KO* cells, FCGBP and UDP-glucuronosyltransferases were highly upregulated (**Table 1**). To elucidate insights into the function of the proteome, gene ontology enrichment analysis showed that WT cells were upregulated for genes associated with temperature stimulus, biosynthetic process, protein stabilization and translation, while *MUC2 KO* cells were highly upregulated for the E-cadherin stabilization pathway (**Figure 1F**). WT cells also showed an increase in biological processes associated with responses to stimulus and metabolic process (**Figure 1G**), which could include responses to mucus secretagogues such as PMA and the highly demanding metabolic process of producing and secreting MUC2 mucin. Using immunofluorescence confocal microscopy, WT goblet-like cells showed ~45% FCGBP that was colocalized with ~80% MUC2 mucin with both proteins abundantly present in the theca at the apical surface of the cell (**Figures 2A, B**). Surprisingly, another pool of FCGBP protein in WT was diffused throughout the cytoplasm not associated with MUC2 (**Figure 2A** top left middle panel). Consistent with the proteome data that showed high FCGBP protein expression in *MUC2 KO* cells (**Table 1**), FCGBP expression was significantly higher (~40%) and diffusely distributed throughout the cytoplasm in *MUC2 KO* as compared to WT cells (**Figures 2C, D**, top right middle panel). These results demonstrate that FCGBP and MUC2 mucin are coordinately regulated in WT goblet-like cells and suggest that the presence of MUC2 influences the expression and secretion of FCGBP. In addition, a free cytoplasmic pool of FCGBP not associated with MUC2 mucin suggests that FCGBP may have diverse functions within the cell.

**Table 1:** The most abundant proteins that were up- and down-regulated between WT and *MUC2 KO* LS174T goblet cells by proteomics analysis.

Gene Name	Protein name	Uniprot ID	Log2(KO to WT)
HIST1H2BN	Histone H2B type 1-N	Q99877	-2.827358878
MUC2	Mucin-2	Q02817	-2.627092465
HIST1H1B	Histone H1.5	P16401	-1.877685101
HSPB1	Heat shock protein beta-1	P04792	-1.830434114
RPL36A	60S ribosomal protein L36a-like;60S ribosomal protein L36a	P83881	-1.31211515
YWHAE	14-3-3 protein epsilon	P62258	-1.273099047
PDXK	Pyridoxal kinase	O00764	-1.198990382
SEC22B	Vesicle-trafficking protein SEC22b	O75396	-1.082621714
CTSD	Cathepsin D	P07339	-0.981280378
GRN	Granulins	P28799	-0.928264785
USP5	Ubiquitin carboxyl-terminal hydrolase 5	P45974	-0.924207262
GMPS	GMP synthase [glutamine-hydrolyzing]	P49915	-0.919369622
PLIN3	Perilipin-3	O60664	-0.916943251
EIF2S1	Eukaryotic translation initiation factor 2 subunit 1	P05198	-0.911071446
ACAA2	3-ketoacyl-CoA thiolase, mitochondrial	P42765	-0.893276481
RAB11A	Ras-related protein Rab-11A;Ras-related protein Rab-11B	P62491	-0.879833883
SMARCE1	SWI/SNF-related matrix-associated actin-dependent regulator of chromatin subfamily E member 1	Q969G3	-0.834904882
GNS	N-acetylglucosamine-6-sulfatase	P15586	-0.833850177
HMGCS2	Hydroxymethylglutaryl-CoA synthase, mitochondrial	P54868	-0.829126338
CTNND1	Catenin delta-1	O60716	-0.807603808
FABP1	Fatty acid-binding protein, liver	P07148	-0.806341785
RCC1	Regulator of chromosome condensation	P18754	-0.801229278
FAM136A	Protein FAM136A	Q96C01	-0.78493029
TMSB10	Thymosin beta-10	P63313	-0.783861802
MRPL43	39S ribosomal protein L43, mitochondrial	Q8N983	-0.772899717
MIF	Macrophage migration inhibitory factor	P14174	-0.76945265
SPR	Sepiapterin reductase	P35270	-0.746615764
NPM3	Nucleoplasmin-3	O75607	-0.745623647
RPS20	40S ribosomal protein S20	P60866	-0.742096274
DNASE2	Deoxyribonuclease-2-alpha	O00115	-0.740552743
ZYX	Zyxin	Q15942	0.685267407
FCGBP	IgGFc-binding protein	Q9Y6R7	0.685357124
GPA33	Cell surface A33 antigen	Q99795	0.69634999
CKB	Creatine kinase B-type	P12277	0.719577745
RBM39	RNA-binding protein 39	Q14498	0.735175542
INF2	Inverted formin-2	Q27J81	0.752149128
UGT1A6	UDP-glucuronosyltransferases	P19224	0.763751492
EPB41L2	Band 4.1-like protein 2	O43491	0.777451278
HSP90AB2P	Putative heat shock protein HSP 90-beta 2	Q58FF8	0.789186704
ATP1A1	Sodium/potassium-transporting ATPase subunit alpha-1	P05023	0.801241448
BSG	Basigin	P35613	0.809002775
CD9	Tetraspanin; CD9 antigen	P21926	0.818686981
DPEP1	Dipeptidase 1	P16444	0.854873369
H1F0	Histone H1.0	P07305	0.866393698
DYNC1I2	Cytoplasmic dynein 1 intermediate chain 2	Q13409	0.918691534
CTNNB1	Catenin beta-1	P35222	0.933497103
S100A11	Protein S100-A11	P31949	0.94732952
SEC61B	Protein transport protein Sec61 subunit beta	P60468	1.000072133
ATP1B1	Sodium/potassium-transporting ATPase subunit beta-1	P05026	1.158983251
IARS2	Isoleucine--tRNA ligase, mitochondrial	Q9NSE4	1.213067271
EPHA2	Ephrin type-A receptor 2	P29317	1.216175457
CTNNA1	Catenin alpha-1	P35221	1.22595354
PSMD2	26S proteasome non-ATPase regulatory subunit 2	Q13200	1.227802676

Blue highlighted proteins are upregulated in WT and in red for MUC2 KO cells. MUC2 and FCGBP are highlighted in yellow for comparison.

### 3.2 Muc2 and Fcgb expression in *Muc2^+/+^* and *Muc2^-/-^* littermates

**Figure 3:**
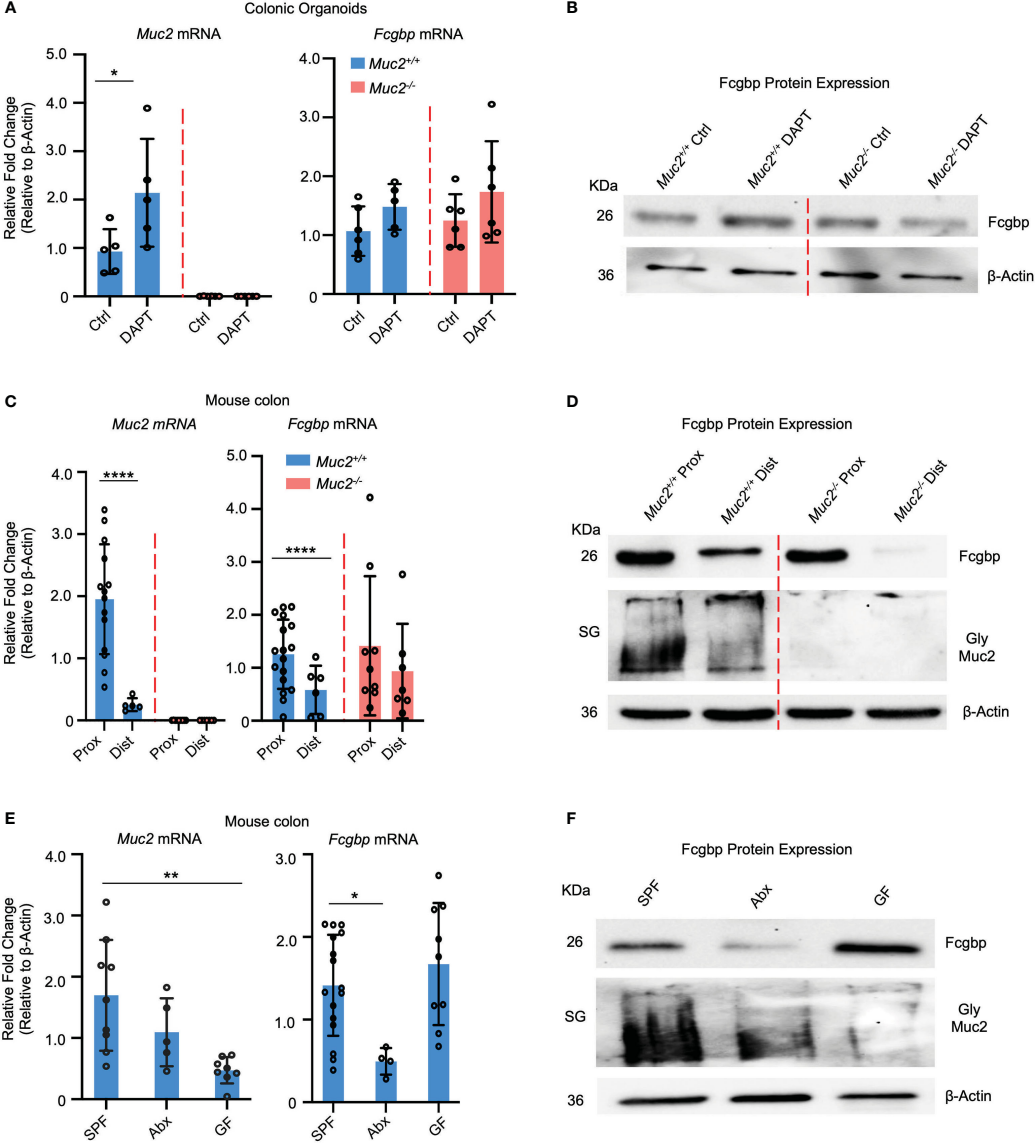
MUC2 and FCGBP expression are coordinately regulated in animals. **(A)** Colonic mouse organoids were isolated and grown basally or in the presence of DAPT to skew cells to a goblet cell phenotype and mRNA expression was measured through RT-PCR (n=5-6). **(B)** Fcgbp protein expression in colonic organoids using Western blot analysis. β-Actin was used as a loading control (n=4). **(C)** mRNA expression from proximal and distal colon tissues of *Muc2^+/+^* and *Muc2^-/-^* littermates. ****p<0.0001 (n=5-10 mice per group). **(D)** Representative Western blot of the expression of Fcgbp and Muc2 in mouse tissues from the proximal and distal colon. β-Actin was used as a loading control (n-5-10). **(E)** mRNA from colonic tissues of SPF mice, antibiotics-treated SPF mice, or GF mice were compared for Muc2 and Fcgbp expression through RT-PCR analysis. *p<0.05, **p < 0.01. SPF, Specific pathogen free; Abx, antibiotics; GF, Germ free; (n=5-10 mice per group). **(F)** Protein expression of Fcgbp and Muc2 in SPF, Abx, and GF proximal colonic tissue through Western blotting analysis. β-Actin was used as a loading control (n=5).

To verify if cultured human goblet-like cells show a similar expression profile for Fcgbp and MUC2 *in vivo*, colonic organoids were generated from *Muc2^+/+^* and *Muc2^-/-^* littermates. To optimize goblet cell lineage, colonoids were pre-treated with DAPT, a NOTCH inhibitor that increases the number of goblet-like cells (30, 31). The *Muc2^+/+^* colonoids differentiated with DAPT showed a significant increase in *Muc2* mRNA and protein expression but not for *Fcgbp* (**Figures 3A, B**). The *Muc2^-/-^* colonoids treated with DAPT showed a slight but not significant increase in *Fcgbp* mRNA expression since these mice retain the presence of goblet-like cells in the absence of Muc2 (32) (**Figures 3A, B**).

To determine if there was a regional difference in basal expression of Muc2 and Fcgbp in the proximal and distal colon of mice, tissues were isolated and analyzed for mRNA and protein expression. We have previously shown that mature goblet cell populations are highest in the proximal colon (33). In accordance, *Muc2* mRNA levels were markedly increased in the proximal but not the distal colon of *Muc2^+/+^* whereas, *Fcgbp* mRNA levels were similar in *Muc2^+/+^* and *Muc2^-/-^* littermates (**Figure 3C**). Predictably, glycosylated Muc2 protein was highly expressed in the proximal as compared to the distal colon in *Muc2^+/+^* littermates (**Figure 3D**). While Fcgbp protein expression was high in the proximal colon of both genotypes, it was absent in the distal colon of *Muc2^-/-^* littermates (**Figure 3D**). Proteomic analysis of proximal/mid colonic tissues revealed that Fcgbp and Clca1 were highly abundant in *Muc2^-/-^*, whereas Muc2 and Fcgbp were abundant in *Muc2^+/+^* littermates (**Table 2** highlighted). These observations indicate that Muc2 and Fcgbp are coordinately regulated throughout the length of the colon with the highest expression in the proximal colon associated with high numbers of goblet cells as compared to the distal colon.

**Table 2:** Proteomics analysis of mucus associated proteins found in mouse colonic tissues and their hit number.

Gene	Protein Name	Uniprot ID	WT	*Muc2 KO*
Muc2	Mucin 2	Q80Z19	24	333
Fcgbp	Fc fragment of IgG-binding protein	Q497G7	29	9
Clca1	Calcium-activated chloride channel regulator 1	Q9D7Z6	34	14
Agr2	Anterior gradient protein 2	O88312	59	40
Zg16	Zymogen granule membrane protein 16	Q8K0C5	248	270
Klk1	Kallikrein 1	P15947	556	356

Higher abundant proteins (highlighted) are represented by a lower number.

As gut microbiota are known to affect goblet cell lineage and Muc2 mucin production and function (25, 26, 34–36), we quantified the expression of Muc2 and Fcgbp in specific-pathogen-free (SPF) animals and those that were either untreated or given broad-spectrum antibiotics (Abx) to reduce microbiota diversity and abundance (25, 37) as well as germ-free (GF) mice. SPF mice treated with Abx showed a significantly decreased *Muc2* mRNA expression and a greater reduction in *Fcgbp* (38–40) (**Figure 3E**). GF mice had very low basal *Muc2* whereas *Fcgbp* mRNA expression was high and showed lower Muc2 protein expression in Abx-treated mice and even lower levels in GF mice. Fcgbp protein expression was similarly decreased in Abx-treated SPF mice whereas basal levels in GF mice were high (**Figure 3F**). Fcgbp was not decreased in GF mice as we hypothesized given that the mucus layer was noticeably thinner and less defined in GF mice (39, 41). As antibiotic treatment decreased Fcgbp in SPF mice, we cannot rule out the possibility that the antibiotics themselves may influence the expression of Fcgbp.

### 3.3 FCGBP is localized within MUC2 mucin granules

**Figure 4:**
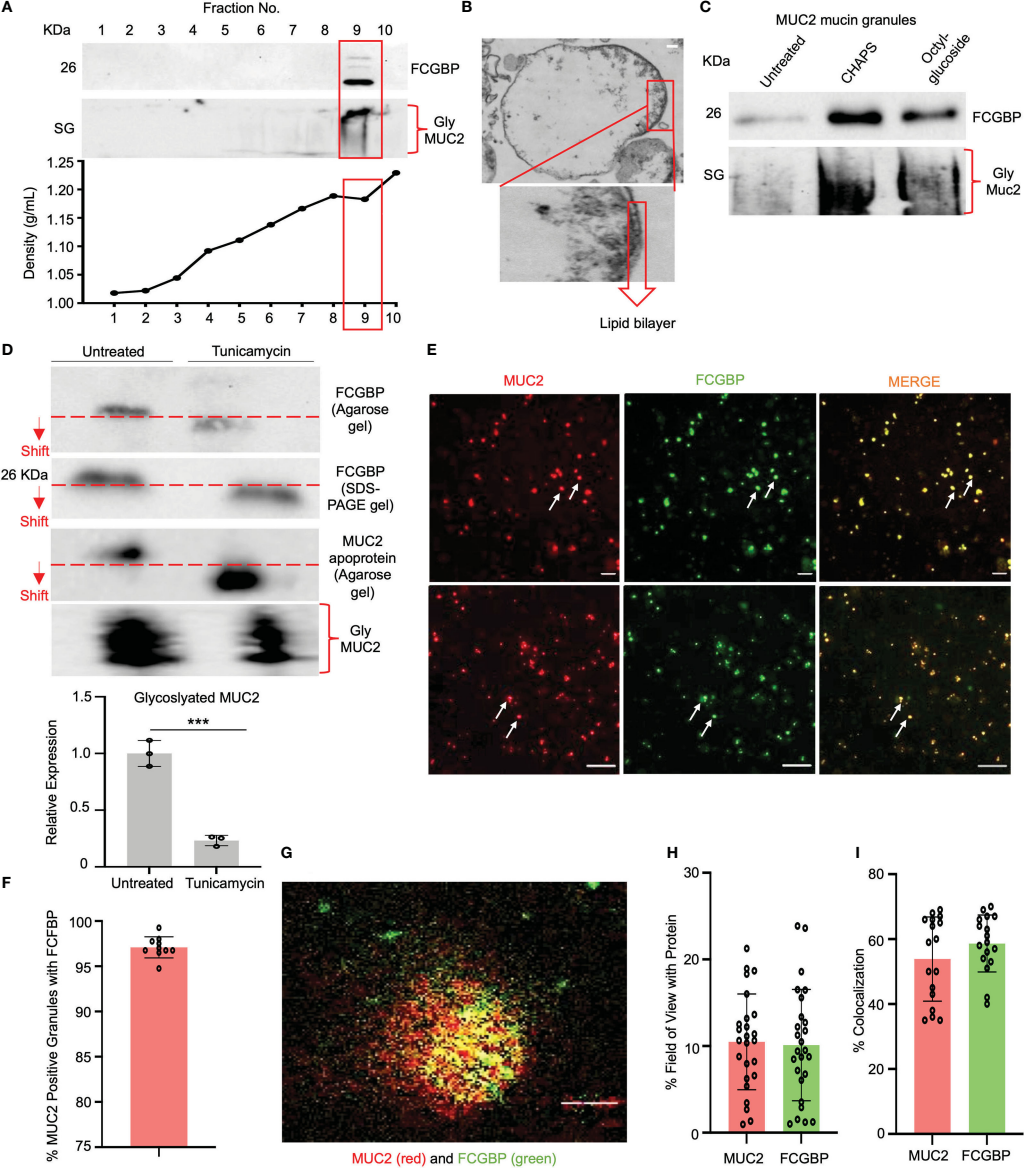
FCGBP is highly enriched in mucin granules from goblet-like cells. **(A)** Western blot analysis of LS174T mucin granules purified by differential centrifugation and sucrose gradient ultracentrifugation. MUC2 and FCGBP were localized in high-density fraction 9. SG = stacking gel. **(B)** Goblet cell mucin granules from A above were visualized by transmission electron microscopy highlighting the lipid bilayer. Inset is a close-up view of the lipid bilayer. Scale bar (top right) = 100 nm. **(C)** Mucin granules treated with weak detergents CHAPS and octyl-glucoside to solubilize the lipid bilayer showed increased immunoreactivity of FCGBP and MUC2. SG = stacking gel. **(D)** Treatment of LS174T goblet cell with the N-linked glycosylation inhibitor tunicamycin caused a shift in MUC2 apoprotein and FCGBP migration pattern on a 1% agarose gel or SDS-PAGE/Western blot, as well as a marked decreased in glycosylated MUC2. ***p<0.001. **(E)** Widefield microscopy images of purified mucin granules stained for MUC2 (red) or FCGBP (green) demonstrating colocalized of both proteins (arrows). Images are representative of 3 separate experiments. Scale bar = 20 μm. **(F)** Quantification of the number of MUC2 positive granules from E that contain FCGBP (n=10). **(G)** STED images of granules isolated from LS174T goblet-like cells stained for MUC2 (red) and FCGBP (green). Images are representative of 3 separate experiments. Scale bar = 0.5 μm. **(H)** Quantification of MUC2 and FCGBP in granules imaged through STED imaging. **(I)** Quantification of colocalization between MUC2 and FCGBP imaged through STED imaging. 24 unique images were used from 3 separate granule preparations.

As MUC2 and FCGBP are highly colocalized in cultured goblet-like cells (**Figure 2A**), this suggests that perhaps both proteins are biosynthesized and packaged together in mucin granules. To determine if these proteins were tightly bound to each other or are spatially separated within mucin granules, we isolated and purified MUC2 mucin granules. To do this, log-phase goblet-like cells (days 5-6) were homogenized, and following a series of differential centrifugation, the enriched mucin granules were isolated using sucrose gradients (**Figure 4A**). The purified granules enriched for FCGBP and the glycosylated MUC2 were partitioned in fraction 9, with a density of ~1.16 – 1.19 g/ml sucrose, suggesting that both proteins were localized with each other. By electron microscopy, purified mucin granules were distinct and irregular shapes, heterogenous in diameter and size (24, 42), and contained a lipid bilayer (**Figure 4B**). Removal of the lipid bilayer with weak detergents, CHAPS, and octyl-glucoside surprisingly increased immunoreactivity of both FCGBP and MUC2 (**Figure 4C**), clearly indicating that both these proteins were localized and shielded within mucin granules. However, the major question still remained: how do FCGBP and MUC2 biochemically interact with each other via glycan-glycan and/or protein-protein interaction within mucin granules? Recent studies have shown that FCGBP is not covalently bound to MUC2 in secreted mucus (16). As interchain disulfide bonds involve several highly conserved, cysteine-rich protein domains that are usually N- but not O-glycosylated (15), we hypothesized that FCGBP interacts via N-linked glycan-glycan interaction with MUC2 C-terminal interchain bonds. Thus, to confirm the importance of N-linked glycans in MUC2 and FCGBP, goblet-like cells were treated with the N-linked glycosylation inhibitor tunicamycin (18) that showed a shift in the migration pattern of the apoproteins on agarose and SDS-PAGE gels cumulating with a significant decrease in total glycosylated MUC2 mucin (**Figure 4D**). To determine if FCGBP and MUC2 were dissociated from each other in mucin granules in the goblet cell theca (as in **Figure 2A** in WT cells), cells were imaged basally and after incubation with tunicamycin. As predicted, FCGBP and MUC2 were significantly dissociated with each other following treatment with tunicamycin and the proteins were dispersed throughout the cytoplasm with no discernable goblet cell theca as compared to untreated controls (**Supplementary Figures 2A, B**). Whole cells lysates from tunicamycin treated cells showed shifts in FCGBP (agarose gel) and MUC2 on both agarose and SDS-PAGE acrylamide gels (**Supplementary Figure 2C**). These results demonstrate that FCGBP and MUC2 probably interacted together via N-linked glycans in mucin granules (**Figure 4D**) and dissociated from the goblet cell theca and dispersed in the cell cytoplasm (**Supplementary Figure 2A**). However, we cannot exclude the possibility that N-linked glycans were also necessary for proper intracellular compartmentalization/sorting of FCGBP and MUC2.

Based on the Western blot data showing that granules are enriched for FCGBP and MUC2 mucin (**Figure 4C**), microscopy was used to visualize the colocalization of the proteins. Using widefield microscopy, ~97% of the granules were positive for MUC2 (red) and FCGBP (green) using fluorescent-labeled antibody-tagged proteins (**Figures 4E, F**). As mucin granules are small (0.5 – 2 µm) (42), the resolution of widefield microscopy was not sufficient to discern the spatial organization of the antibody-tagged proteins. To resolve this, we used stimulated emission depletion (STED) super-resolution microscopy to visualize and image the proteins in granules with a high resolution of approximately 30-50 nm (43) vs the resolution of confocal microscopy. STED microscopy revealed that MUC2 (red) and FCGBP (green) proteins were randomly distributed within a granule, were heterogeneous, and not all granules had the same localization of each protein (**Figures 4G, H**). However, in most granules, MUC2 and FCGBP proteins were spatially colocalized with each other (53% and 58%, respectively; **Figure 4I**). These results suggest that both MUC2 and FCGBP are highly colocalized in heterogenous subpopulations of mucin granules.

### 3.4 Quantitative proteomic analysis of mucin granules

**Figure 5:**
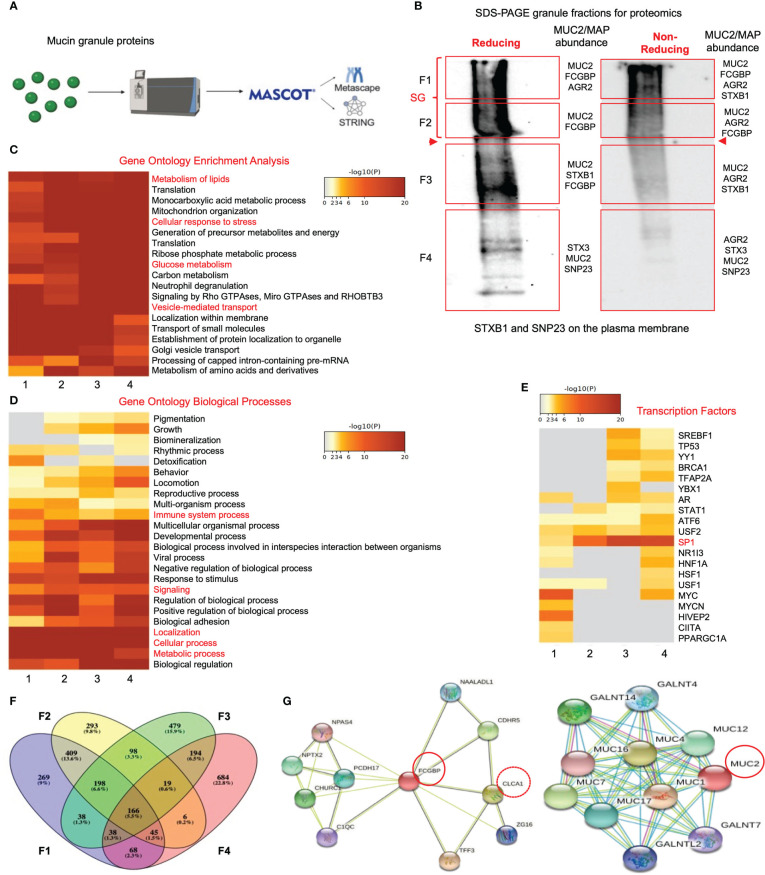
Proteomic analysis of purified human mucin granules from LS174T cells under reducing and non-reducing conditions. **(A)** Shotgun proteomic analysis workflow. **(B)** Western blot of mucin granules run on SDS-PAGE under reducing and non-reducing conditions and probed with an antibody for glycosylated MUC2. The red-boxed areas designated as fractions F1 to F4 were excised for proteomic analysis. MUC2/MAP (mucus-associated protein) protein of interest for each fraction is shown on the right-hand side of each box. Bold red arrowheads indicate the junction between the stacking gel (SG) and the resolving gel. **(C)** Metascape analysis showing gene ontology enrichment of pathways utilized by each fraction under reducing conditions. Pathways of interest are highlighted in red. **(D)** Metascape analysis showing gene ontology biological processes associated with the proteins present in each of the 4 fractions under reducing conditions. Pathways of interest are highlighted in red. **(E)** Metascape analysis showing transcription factors associated with protein hits from each mucin granule fraction under reducing conditions. Transcription factors of interest are highlighted in red. **(F)** Venn diagram showing overlapping and unique proteins from each of the 4 fractions. The Venn diagram was made with Venny (44). **(G)** STRING analysis showing known protein-protein interactions for FCGBP and MUC2.

To gain insights into the functional interactions between FCGBP and MUC2 and other putative granule proteins, purified mucin granules were electrophoresed by SDS-PAGE under reducing and non-reducing conditions, and the global proteome of the different molecular weight fractions was resolved using quantitative shotgun proteomic analysis (**Supplementary Table 2**, **Figures 5A, B**). Reducing and non-reducing conditions were used to maximize the localization of the MAPs that could interact with MUC2 by disulfide bonding. Proteins of interest related to MUC2 mucin, MAPs, and proteins involved in mucus exocytosis were found in all 4 fractions in reducing and non-reducing gels (**Figure 5B**, **Table 3**). Predictably, MUC2 was abundant in fractions 1 and 2 (stacking gel; SG) in reducing and non-reducing gels but was also found throughout the other 2 fractions in the resolving gel. AGR2, which plays a role in MUC2 assembly, folding, and trafficking, was located throughout the gel under non-reducing conditions. Similarly, the SNARE proteins, STXB1, STX3, and SNP23 which are associated with MUC2 mucin exocytosis (24, 45), were located throughout the gel. Importantly, FCGBP was present in fractions 1-3 under reducing conditions and in fractions 1 and 2 under non-reducing conditions. The higher abundance of FCGBP and MUC2 in fractions 1 and 2 (stacking gel) under non-reducing conditions suggests that the interaction between these proteins was easily disrupted by reducing agents. Importantly, of all the MAPs, only FCGBP and AGR2 were identified in purified granules by proteomic analysis. These findings highlight the abundance of FCGBP/MUC2 packaged within mucin granules.

**Table 3:** Attractive protein hits from the proteomic analysis of purified mucin granules derived from human LS174T goblet cells.

Gene Name	Protein name	Uniprot ID	Reducing Fraction No.	Non-Reducing Fraction No.
MUC2	Mucin 2	Q02817	1, 2, 3, 4	1, 2, 3, 4
FCGBP	IgG Fc binding protein	Q9Y6R	1, 2, 3	1, 2
AGR2	Anterior gradient 2	O95994	1	1, 2
STXB1	Syntaxin binding protein 1	P61764	3	3
STX3	Syntaxin 3	Q13277	4	4
SNP23	Snap 23	O00161	4	4
MUC5AC	Mucin 5AC	P98088	1, 2	2
MUC5B	Mucin 5B	Q9HC8	1, 2	2
MUC6	Mucin 6	Q6W4X	1, 2, 3	2, 3, 4
MUC13	Mucin 13	Q9H3R	1, 2, 3, 4	1, 2, 3, 4
MUC17	Mucin 17	Q685J3	1	–
CDHR5	Cadherin-related family member 5	Q9HBB	1, 2	2
GALNT1	N-acetylgalactosaminyltransferase 1	Q10472	1, 2, 3	1, 2, 3
GALNT2	N-acetylgalactosaminyltransferase 2	Q10471	1, 2, 3	1, 2, 3, 4
GALNT3	N-acetylgalactosaminyltransferase 3	Q14435	3	3
GALNT4	N-acetylgalactosaminyltransferase 4	Q8N4A	3	3, 4
GALNT5	N-acetylgalactosaminyltransferase 5	Q7Z7M	1, 2	1, 2, 3
GALNT6	N-acetylgalactosaminyltransferase 6	Q8NCL	3	–
GALNT7	N-acetylgalactosaminyltransferase 7	Q86SF2	1, 3, 4	1, 2, 3, 4
GALNT10	N-acetylgalactosaminyltransferase 10	Q86SR1	3	3
GALNT12	N-acetylgalactosaminyltransferase 12	Q8IXK2	3	–
GALNT13	N-acetylgalactosaminyltransferase 13	Q8IUC8	3	2
B3GALNT2	β-1,3-N-acetylgalactosaminyltransferase	Q8NCR	3	–
MGAT1	α-1,3-mannosyl-glycoprotein 2-β-N-acetylglucosaminyltransferase	P26572	4	4
MGAT2	α-1,6-mannosyl-glycoprotein 2-β-N-acetylglucosaminyltransferase	Q10469	3, 2	–
MGT4A	α-1,3-mannosyl-glycoprotein 4-β-N-acetylglucosaminyltransferase	Q9UM2	3	–
MGT5A	α-1,6-mannosylglycoprotein 6-β-N-acetylglucosaminyltransferase	Q09328	3	2, 3
MPST	3-mercaptopyruvate sulfurtransferase	P25325	4	4
ERP29	Endoplasmic reticulum resident protein 29	P30040	1, 2, 3, 4	1, 2, 3, 4
TAGL2	Transgelin-2	P37802	–	1, 4
S100a6	Protein S100-A6	P14069	–	3
PIGR	Polymeric immunoglobulin receptor	P01833	1, 2, 3, 4	1, 2, 3
RNH1	Ribonuclease inhibitor	P13489	4	4
PSMD2	26S proteasome non-ATPase regulatory subunit 2	Q13200	3	1, 2, 3
ELAVL1	ELAV-like protein 1	Q15717	4	1, 3, 4
CTSD	Cathepsin D	P07339	4	1, 2, 3, 4
DCN	Decorin	P07585	3	3
FAM3D	Protein FAM3D	Q96BQ	4	1, 2, 3, 4
GPA33	Cell surface A33 antigen	Q99795	1, 2, 3, 4	1, 2, 3
LGALS3	Galectin-3	P17931	4	4
LGALS3BP	Galectin-3 binding protein	Q08380	1, 2, 3, 4	1, 2, 3,4

To gain insights into the functions of the mucin granule proteome, the data from reducing (**Figures 5C, G**) and non-reducing (**Supplementary Figures 3A, D**) gels were analyzed for pathway and gene ontology enrichment and protein network using meta-analysis (20) (metascape.org). All 4 fractions showed enrichment in proteins that were involved in high-metabolic activities such as metabolism of lipids, cellular response to stress, glucose metabolism, and vesicle-mediated transport (**Figure 5C**). More specifically, these proteins were associated with metabolic and cellular processes as well as biological processes related to immune system processes and signaling (**Figure 5D**). The gene ontology pathway data demonstrated that the mucin granule proteins are complex, consisting of proteins that are related to a range of processes and functions associated with high metabolic activity. In addition to the gene ontology pathway analysis, the transcription factor for MUC2, SP1 (46), was highly associated with mucin granule proteins (**Figure 5E**). The distribution of the proteins analyzed through a Venn diagram showed overlap of proteins between each of the 4 fractions (**Figure 5F** *).* This was consistent with the meta-analysis findings that showed enrichment of proteins in all 4 fractions involved in metabolic and cellular processes and transcription factors. Importantly, MUC2 was present in all 4 fractions under both reducing and non-reducing conditions, whereas FCGBP was present in fractions 1-3 in reducing and fractions 1-2 in non-reducing gels.

To elucidate the functional interactions between MUC2 and FCGBP and other MAPs, we used STRING-db v11 analysis database (21) (https://string-db.org). Strikingly, only FCGBP showed multiple interactions with the other MAPs including CLCA1, ZG16, and TFF3. ARG2, CLCA1, ZG16, and KLK1 did not interact with each other (**Supplementary Figure 3D**). Other protein interactions that were identified included the transcription factor NPAS4, adhesion molecules (PCDH17 and CDHR5) and the complement-associated proteins C1QC (**Figure 5G**). The MUC2 protein interactions were predominantly with MUC12, MUC4, MUC16, MUC7, MUC1 and MUC17 (**Figure 5G**). Additionally, numerous polypeptide N-acetyl-galactosaminyltransferases (GALNT14, GALNT4, GALNT2, and GALNT7) known to be associated with mucin glycosylation were identified (39) (**Table 3**). Importantly, no MAPs other than FCGBP interacted with MUC2 by STRING analysis. Other proteins of interest identified from the proteomic data (**Table 3**) include CDHR5, an important adhesion molecule between microvilli and enterocytes of the intestine (47). Additionally, multiple N-acetylgalactosaminyltransferases were identified (GALNT1, GALNT2, GALNT3, GALNT4, GALNT5, GALNT6, GALNT7, GALNT10, GALNT12, GALNT13 and B3GALNT2). This family of glycosyltransferases is important in the O-glycosylation of mucins such as MUC2 (48). N-Acetylglucosaminyltransferases were also found including MGAT1, MGAT2, MGT4A, and MGT5A. This subgroup of acetyl-glucosaminyltransferases is associated with N-linked glycans in organelles throughout the body (49, 50). Of particular interest were proteins that had previously been identified in secreted mucus from mouse and human studies and determined to be closely related to Muc2 through proteomic analysis (11, 51). Indeed, several proteins that were identified in secreted mucus throughout the GI tract were identified in the mucin granules (**Table 3**) including ERP29, TAGL2, S100a6, PIGR, RNH, PSMD2, ELAVL1, CTSD, DCN, FAM3D, GPA33, LGALS3, and LGALS3BP. While no functional significance has been ascribed to these proteins within mucin granules or secreted mucus, it was of interest to observe the overlap of proteins within granules and in secreted MUC2 mucus. Taken together, the proteomic data highlight the complex nature of the mucin granules and demonstrate that the contents contain numerous proteins known to be associated with secreted MUC2 mucus.

### 3.5 FCGBP is non-covalently bound to MUC2 in secreted mucus

**Figure 6:**
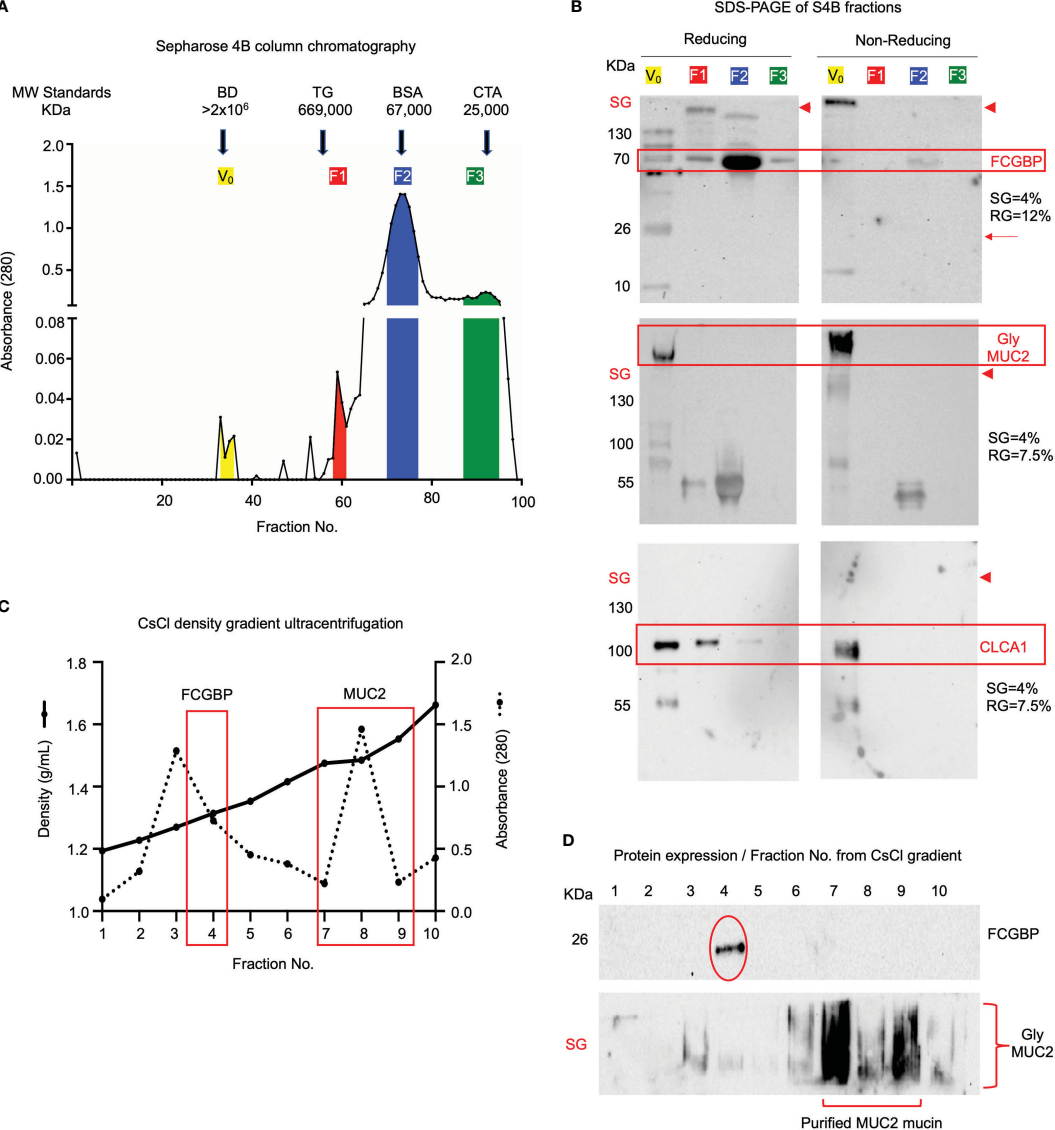
FCGBP is found in secreted mucin from LS174T goblet-like cells and is non-covalently attached to MUC2. **(A)** Sepharose 4B (S4B) column chromatography of native LS174T secreted mucus. The colored sections represent different molecular weight fractions from S4B and were probed using Western blotting for MUC2 and FCGBP. Arrows indicate the location of known molecular weight standards: Blue Dextran (BD: >2x10^6^ KDa), Thyroglobulin (TG: 669 KDa), Bovine Serum Albumin (BSA: 67 KDa), and Chymotrypsinogen A (CTA: 25 KDa). **(B)** S4B fractions ran under reducing and non-reducing conditions for Western blot analysis. Blots were immunoblotted with antibodies for FCGBP, MUC2 glycosylated proteins, and CLCA1. FCGBP separated from MUC2 under reducing conditions but remains intact in the V_0_ stacking gel (SG) under non-reducing conditions. Major protein bands of interest are highlighted in red. The red arrowheads indicate the junction between the stacking gel (SG) from the running gel. SDS-PAGE gels probed for FCGBP consisted of 4% stacking gel and 12% running gel and for MUC2 and CLCA1, 4% stacking gel and 7.5% running gel. **(C)** CsCl density ultracentrifugation of MUC2 V_o_ mucin isolated by S4B in A above. The location of the low-density fraction where FCGBP partitioned and the high-density fractions that contain MUC2 are boxed in red. **(D)** Western blot analysis for FCGBP and MUC2 for each of the CsCl density ultracentrifugation fractions. Note that MUC2 is present in the high-density fractions 5-8, whereas FCGBP is only present in the lower-density fraction 4. SG, stacking gel.

As MUC2 and FCGBP were shown to be abundantly expressed, packaged, and localized in mucin granules, we next investigated how these proteins interact and partition in secreted native polymeric mucus gels. To determine if FCGBP and MUC2 were covalently attached to each other (15), secreted mucus from LS174T spend culture media was purified under native conditions (Tris-HCL buffer, pH 8.0) using Sepharose 4B (S4B) column chromatography as previously described (52). High molecular weight MUC2 mucin eluted in the void volume (V_0_) (**Figure 6A**, fractions 29–36) whereas other non-mucin proteins eluted at different molecular weights fractions designated F1, F2, and F3 (color coded). To visualize the proteins, S4B protein fractions separated by SDS-PAGE (**Figure 6B**) under reducing [left panels, 4% stacking gel (SG) and 12% resolving gel (RG)] and non-reducing [right panel, 4% stacking gel (SG) and 7.5% resolving gel (RG)] conditions revealed that the majority of FCGBP and MUC2 were bound to each other (red boxed area). Under non-reducing conditions, FCGBP was present with MUC2 in the stacking gel (SG; right panels above the red arrowheads), and under reducing conditions, FCGBP separated into several protein fractions with two major bands at 70 and 26 KDa (left panels). Under reducing conditions, most of the 70 KDa FCGBP was unbound with MUC2 and was present in all fractions but was abundant in the low molecular weight F2 fractions (Fractions 70-77). Under non-reducing conditions, the majority of FCGBP and MUC2 were present together in the stacking gel suggesting a role for disulfide bonds between these proteins in secreted mucus. However, STRING-db analysis revealed no protein-protein interactions between FCGBP and MUC2. Instead, FCGBP showed direct interaction with other MAPs including CLCA1 (**Figure 5G**, stippled circle), and indeed, blots probed for CLCA1 showed its presence with FCGBP in V_0_ under reducing and non-reducing conditions (**Figure 6B**, third panel). To elucidate if MUC2 and FCGBP were covalently bound, V_0_ MUC2 mucin from S4B was purified using cesium chloride (CsCl) density gradient ultracentrifugation that separates covalently from non-covalently bound proteins (52–54). Quantification of the CsCl density gradient fractions (**Figure 6C**) revealed that FCGBP partitioned exclusively in the low-density fraction 4 (1.31 g/mL) as a 26 KDa protein whereas, MUC2 was present in the high-density fractions 7-9 (1.48-1.58 g/mL) and remained in the stacking gel (SG, **Figure 6D**). These findings show that FCGBP and MUC2 are not covalently bound in secreted mucus, contrary to previous speculations (15). Based on this unique interaction, FCGBP-MUC2 binding is probably destabilized in the gut by different pH levels and reducing agents similar to the conditions that occur in acute/chronic inflammation in infectious and inflammatory bowel disease (55, 56).

**Figure 7:**
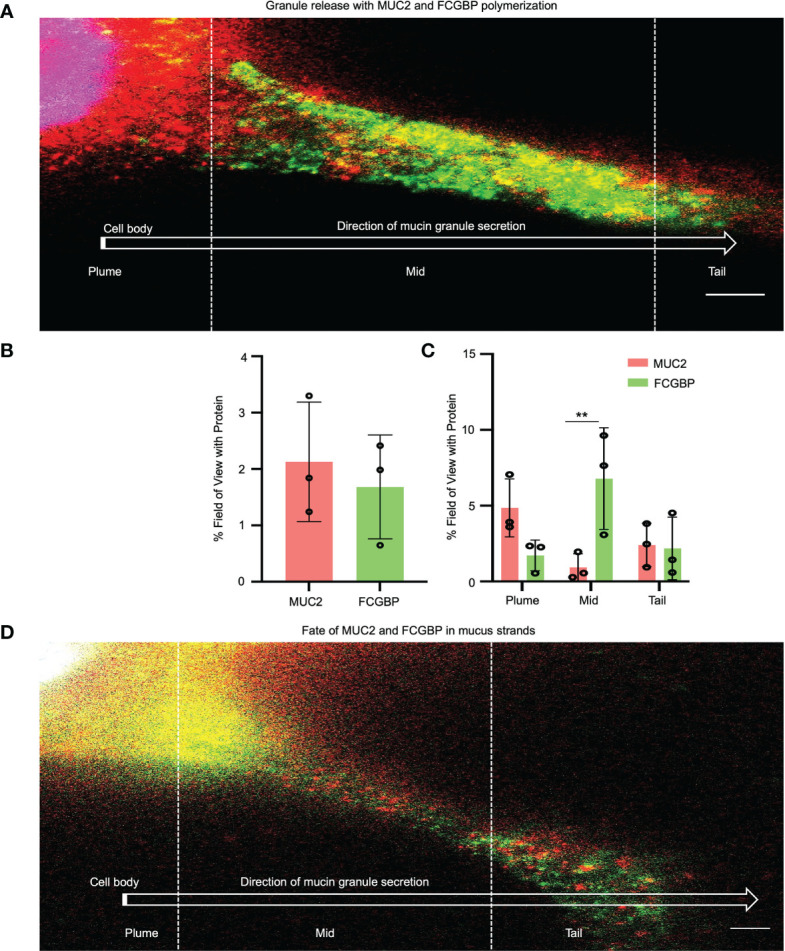
Immunolocalization of MUC2 and FCGBP in secreted mucus stands from LS174T goblet-like cells. **(A)** STED imaging of goblet-like cells secreting MUC2 mucin (red) and FCGBP (green) showing granule release and the polymerization of MUC2 and FCGBP towards the tail region. Scale bar = 1 μm. Images are representative of 3 independent experiments. **(B)** Quantification of total MUC2 and FCGBP in secreted mucin through STED imaging. **(C)** Quantification of MUC2 and FCGBP in the flare, mid, and tail sections of the mucus strand. Images are representative of 3 independent experiments. **p < 0.01. **(D)** STED imaging of LS174T cells showing the fate of MUC2 and FCGBP in mucus strands. Scale bar = 1 μm. Images are representative of 3 independent experiments.

To quantify how MUC2 and FCGBP in mucin granules partition and merge with each other when secreted constitutively from LS174T cells to form mucus strands, STED immunofluorescence microscopy was used to follow the granule contents (**Figure 7**). Cells that were actively secreting mucus were probed with fluorescent-labeled antibodies for MUC2 (red) and FCGBP (green) and a fluorescent stain for DAPI (blue). Newly secreted mucus strands from the plume (near the cell surface, nuclei stain blue) to the tail were clearly visible that showed the spatial distribution of FCGBP and MUC2 (**Figures 7A, B**). Regionally, the mucus strand showed that FCGBP (**Figure 7A**, green) was predominant in the mid-body, whereas MUC2 (red) was highest at the cell junction with lower quantities of FCGBP (**Figures 7A, C**). At the tail region, the granule proteins lost their structure with granule coalescence to form red mucus strands. The fate of FCGBP and MUC2 in older mucus strands (**Figure 7D**) lost their organization and contained abundant aggregates of MUC2 and FCGBP that were either loosely attached to the cell body or were being pinched off the cell body (as shown). These data demonstrate that FCGBP and MUC2 mucin are secreted together and play a critical role in the spatial organization of the mucus strand.

### 3.6 Functional roles of MUC2 bound to FCGBP and cytoplasmic FCGBP in epithelial restitution

**Figure 8:**
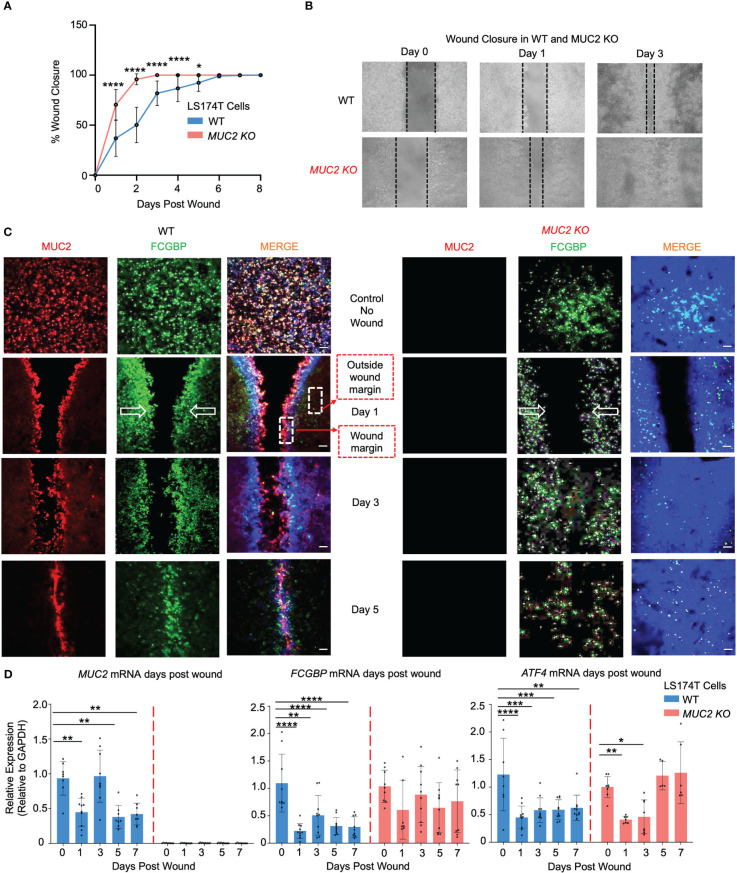
MUC2 and FCGBP expression during restitution in LS174T WT and *MUC2KO* goblet-like cells. **(A)** WT and *MUC2KO* cells were grown with a wound insert leaving a gap of 500 μm. At 0 to 7 days post-wound, the space between the cells was measured to quantify restitution. *p<0.05, ****p<0.0001 (n=6 per day). **(B)** Representative bright field images of the restitution assay. The solid black dotted lines represent the cell front during wound healing. **(C)** Confocal microscopy images of control WT and *MUC2KO* during restitution showing MUC2 (red), FCGBP (green), and DAPI (blue). The open arrows show the direction of cell migration. The stippled white boxed area shows the wound margin and outside of the wound margin that was quantified to enumerate partitioning of MUC2 and FCGBP during wound healing. Images are representative of 3 different experiments. **(D)** At 0 to 7 days post-wound, MUC2, FCGBP, and ATF4 mRNA were analyzed by RT-PCR. GAPDH was used as a housekeeping gene. *p<0.05, **p<0.01, ***p<0.001, ****p<0.0001 (n=6 per day).

As both MUC2 and FCGBP were packaged in mucin granules and were secreted together to form the mucus layer, it was of interest to determine how these proteins interacted in wounded cells and in restitution. To do this, WT and *MUC2 KO* goblet-like cells were plated into 2-well silicone inserts with a defined 50 μm cell-free gap. After the cells became confluent (2 days), the insert was removed, and the cells were allowed to migrate to close the wound gap (open white arrows). Strikingly, he wounds were completely healed by day 2 in *MUC2 KO* but took until day 6 to heal in WT cells (**Figures 8A, B**). To visualize the partitioning of MUC2 and/or FCGBP during restitution, the cells grown without an insert acted as basal controls and following the removal of the insert, the cell fronts were imaged using confocal microscopy on days 1, 3, and 5. Basally, both MUC2 and FCGBP were colocalized in WT cells, whereas FCGBP was diffusely present throughout the cytoplasm in *MUC2 KO* cells (**Figure 8C**, quantification in **Supplementary Figure 4A**). Unexpectedly, once wounded, MUC2 and FCGBP were both highly polarized at the wound margin of WT cells and followed the leading edge of the cells until wound closure at day 6. In contrast, FCGBP unbound to MUC2 in *MUC2 KO* cells did not partition to the leading edge of the wound, suggesting that cytoplasmic FCGBP might play a role in restitution in the absence of MUC2 that healed within 2 days. The evidence for this was that the expression of *MUC2* and *FCGBP* mRNA were significantly decreased in WT cells during restitution, whereas *FCGBP* mRNA expression remained high and unaltered in *MUC2 KO* cells (**Figure 8D**). In both cell types, the stress response gene *ATF4* decreased significantly in wounded cells but returned to control levels upon wound closure only in *MUC2 KO* cells on days 5-7 (**Figure 8D**). Wound healing was mediated primarily by cell migration as the secretory cell lineage transcription factors *MATH1* and *SPDEF* were significantly downregulated post wound (**Supplementary Figure 4B**).

**Figure 9:**
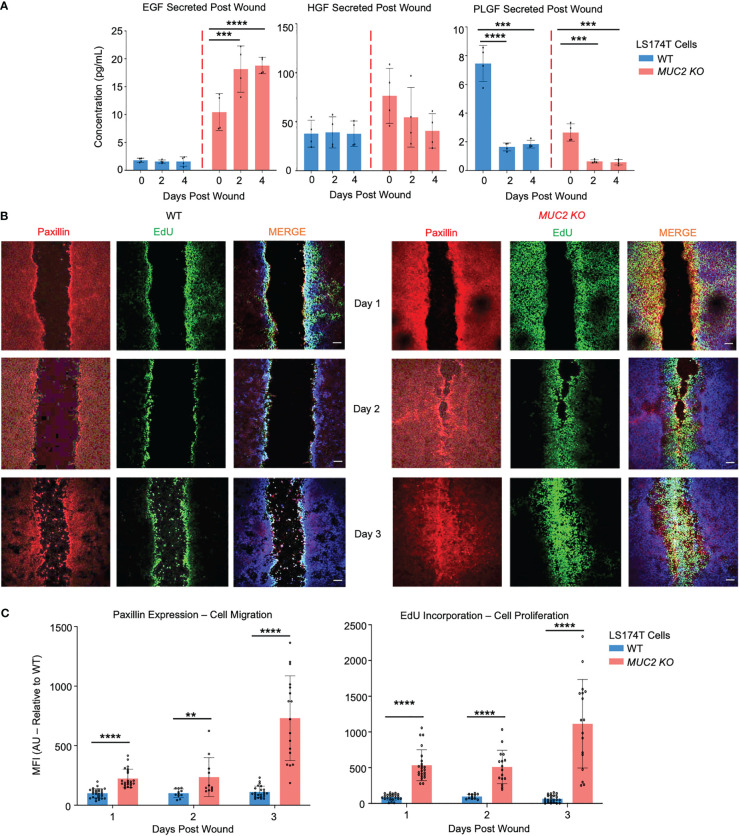
FCGBP accelerates wound healing through growth factors, migration, and proliferation. **(A)** Culture media were collected on day 0 (removal of insert) and on days 2 and 4 after wound insert removal. Samples were analyzed for growth factor production using a Luminex multiplex assay. ***p<0.001, ****p<0.0001 (n=4 per day). **(B)** Confocal microscopy images of control WT and *MUC2KO* during restitution showing paxillin (red), EdU (green), and DAPI (blue). Images are representative of 3 different experiments. Scale bar = 10 μm. **(C)** Quantification of mean fluorescence intensity (MFI) of paxillin and EdU compared to WT. **p<0.01, ****p<0.0001 (n=15).

To gain mechanistic insights into why *MUC2 KO* cells healed within 2 days, secreted growth factors were assessed by a multiplex assay that showed significantly high levels of epidermal growth factor (EGF) in *MUC2 KO*, but not in WT cells; other growth factors (HGF and PLGF) did not play a role in wound healing (**Figure 9A**). To specifically determine if the altered rates of restitution were caused by differences in cell migration, proliferation, or both, wound closure was quantified using confocal microscopy on the wounded cells on days 1, 2, and 3. Cell migration was quantified by staining for the focal adhesion protein paxillin (57) and proliferation was measured by 5-ethynyl-2’-deoxyuridin (58) (EdU) incorporation. As predicted, there was a significant increase in paxillin and EdU incorporation within 2 days that was pronounced by day 3 upon wound closure in *MUC2 KO* as compared to WT cells (**Figures 9B, C**). Cell migration and proliferation are the key cellular processes required for wound closure and healing (59). In WT cells, the presence of FCGBP bound to MUC2 at the wound margin associated with significantly less EGF growth factor secretion, cell migration, and proliferation strongly suggests that cytoplasmic-free FCGBP in *MUC2 KO* cells played a significant role in wound healing.

To assess if the phenomenon we observed in human LS174T goblet-like cells was true of more complex and relevant diseases, the expression of MUC2/FCGBP was determined from human biopsy samples taken from healthy controls and patients with active ulcerative colitis (UC) and UC in remission (UC-R). In active UC patients, *MUC2* and *FCGBP* were significantly upregulated 25-fold and 3-fold, respectively (**Supplementary Figure 4C**), and in UC patients in remission (UC-R), *MUC2* and *FCGBP* expression were similar to healthy controls. These findings suggest that *MUC2* and *FCGBP* are highly regulated during active disease and restitution. Taken together, these data suggest that MUC2 and FCGBP are coordinately altered during epithelial wounding/restitution and in the absence of MUC2, cytoplasmic FCGBP accelerates wound healing.

### 3.7 Fcgbp expression is rapidly altered in DSS colitis in mice

**Figure 10:**
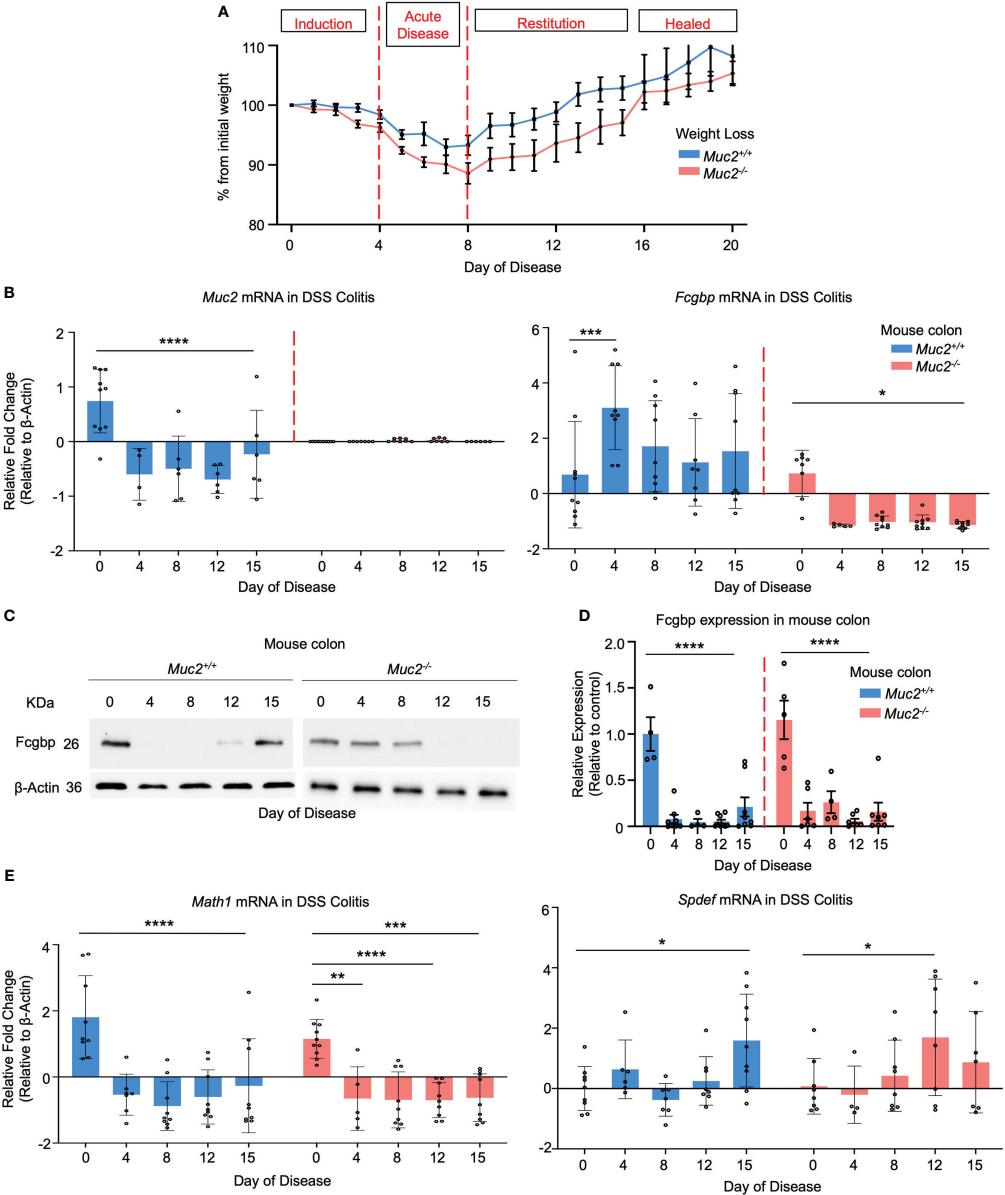
Fcgbp expression is altered in DSS colitis in mice. **(A)** Weight loss in *Muc2^+/+^* (blue) and *Muc2^-/-^* (red) littermates weighed daily after exposure to DSS in their drinking water during acute disease and in restitution. Shown are the percent weight loss/gain from day 0 (n=8-12 mice per day). **(B)** *Muc2* and *Fcgbp* mRNA expression during the 15-day duration of the experiment. β-Actin was used as a housekeeping gene. *p<0.05, ***p<0.001, ****p<0.0001 (n=8-12). **(C)** Fcgbp protein expression in colonic tissues taken during the 15-day duration of the experiment. β-Actin was used as a housekeeping gene. **(D)** Quantification of protein from Western blot analysis during the 15-day duration of the experiment. Protein levels were compared to their own control (day 0). ****p<0.0001 (n=8-12). **(E)** *Math1* and *Spdef* mRNA expression during the 15-day duration of the experiment. β-Actin was used as a housekeeping gene. *p<0.05, **p<0.01, ***p<0.001, ****p<0.0001 (n=8-12).

To determine if Muc2 and Fcgbp are coordinately regulated in an animal model of UC, *Muc2^+/+^* and *Muc2^-/-^* littermates were given DSS in their drinking water to induce colitis and proximal colonic tissues were collected for RT-PCR and Western blot analysis from days 0 to 15 post-DSS as previously described (26, 60). For consistency, we used the same batch and dosage of DSS as our previous study (26). Weight loss was used as an indicator of disease activity associated with scores for stool consistency, blood in stool, and overall animal appearance in both genotypes. Histologically, tissue damage was associated with a thickened muscle layer, loss of crypt architecture, and cellular infiltration throughout the length of the colon, with most damage occurring distally. In both genotypes, colitis was associated with maximal weight loss from days 4-8 (acute disease) before returning gradually to basal weight by day 17 (healed lesions) post-DSS (**Figure 10A**). In *Muc2^+/+^* littermates, *Muc2* expression was significantly decreased as early as day 4 post-DSS (induction phase) and remained below basal levels throughout to day 15 (**Figure 10B**) as shown previously (26, 61). In marked contrast, *Fcgbp* mRNA expression increased significantly by day 4 and remained elevated up to 15 days post-DSS (**Figure 10B**). In *Muc2^-/-^* littermates, even though *Fcgbp* mRNA expression was significantly decreased as early as 4 days post-DSS and remained low up to day 15, colonic protein steadily decreased and was undetectable on days 12-15 post-DSS. Fcgbp protein expression in *Muc2^+/+^* was undetectable as early as day 4 post-DSS but returned to basal levels by day 15 post-DSS during restitution (**Figures 10C, D**). To determine if goblet cell lineage affected the expression of Muc2 and Fcgbp during acute disease and restitution, we quantified the expression of the secretory cell lineage *Math1* and it was significantly downregulated as early as day 4 and remained low up to day 15 post-DSS (**Figure 10E**). As Math1 affects all secretory lineage, we analyzed *Spdef* expression, the transcription factor critical for terminal goblet cell differentiation and observed a steady increase in *Spdef* expression during restitution especially on days 12 and 15. As DSS can adversely affect shifts in microbiota abundance (25, 26) and microbiota can influence Muc2 and Fcgbp mRNA and protein expression (**Figure 3F**) that are critical in wound healing, these findings are not comparable to what was observed in cultured LS174T cells. Nonetheless, these results suggest that the restoration of Fcgbp in *Muc2^+/+^* littermates on day 15 perhaps played an overlooked role in mucosal healing.

## 4 Discussion

The mucus layer is critical to innate host defense against a wide range of pathogens and toxins that it encounters in the gastrointestinal tract. This awareness has been increased in recent years given the central role the MUC2 mucus layers play in sustaining and nourishing indigenous microbiota essential for intestinal homeostasis and health. Embedded in secreted mucus are MAPs and it is unclear how they interact biochemically with MUC2 to convey and enhance mucus protective barrier functions. In this study, we characterized the molecular interaction and functional role of one MAP, FCGBP, as it is a large and heavily glycosylated molecule structurally similar to MUC2. Here we revealed using gene and protein expression studies and biochemical analysis that FCGBP and MUC2 are coordinately biosynthesized, non-covalently bound, packaged with MUC2 mucin in granules, and are secreted to form a structural component of the mucus layer. Importantly, using proteomics, STRING-db v11 analysis, and Western blots, we revealed that FCGBP, coupled with CLCA1, interacted with MUC2 mucin. Biochemical studies revealed that MUC2 and FCGBP are non-covalently bound and are held together via N-linked glycans in secreted mucus. Using confocal microscopy we revealed two pools of FCGBP; one pool was bound to MUC2 in mucin granules and another pool was free in the cytoplasm in cultured goblet-like cells. In wounded WT goblet-like cells, MUC2/FCGBP proteins were highly polarized at the wound margin and played a cooperative role in slow wound healing over 6 days. In marked contrast, in wounded CRISPR-Cas9 gene-edited *MUC2 KO* cells, cytoplasmic FCGBP were diffusely distributed, and wounded cells healed within 2 days. This was associated with high levels of the growth factor EGF. Restitution from DSS colitis in WT mice was associated with increased expression of Fcgbp. To our knowledge, this is the first report to ascribe a functional cooperative role for FCGBP and MUC2 mucin in mucosal protection and restitution.

MUC2 mucin is secreted under basal conditions constitutively and is markedly upregulated in response to mucus secretagogues and inflammatory agents through stimulated release (24, 61–63). Constitutive mucus secretion ensures that there is a basal functional mucus layer throughout the GI tract, whereas stimulated release evokes high-output mucus secretion in response to pathogens and noxious agents to limit contact with the underlying mucosal epithelial cells. The observation that both FCGBP and MUC2 mRNA and protein expression were moderately upregulated basally and inducible in response to the mucus secretagogue PMA suggests that FCGBP is an integral part of the mucus layer. PMA has been extensively shown to upregulate MUC2 expressed through the PKC pathway (29, 42, 64) through its interaction with transcriptional factors such as *SP1* (65). Indeed, both MUC2 and FCGBP share transcription factors such as *SP1*, as well as other transcription factors including *CREB1*, *MYC*, and *ATF2* (66), providing additional evidence for the co-regulation and expression of MUC2 and FCGBP. Interestingly, in LS174T goblet-like cells, stored MUC2 mucin was colocalized with FCGBP in the theca around the nucleus. However, only approximately 50% of FCGBP was colocalized with MUC2 and the rest diffused and distributed throughout the cell. This suggests that there is perhaps a secondary function for FCGBP that is independent of mucus within goblet-like cells. Our results suggest that cytoplasmic FCGBP may play a role in accelerating wound healing in *MUC2 KO* goblet-like cells. In mice, Fcgbp was strongly co-expressed with Muc2 in the proximal colon that contain the highest number of goblet-like cells as compared to the distal sections (33). An unexpected finding was that Abx-treated SPF mice showed significantly decreased expression of Fcgbp, whereas, in GF mice, expression of Fcgbp was high. Abx treatment in SPF mice is known to decrease the expression of Muc2 and may negatively affect the expression of Fcgbp (38–40). As GF mice have decreased Muc2 mRNA expression and a thin mucus layer (41), these findings suggest that reduced Muc2 dysregulated Fcgbp expression, analogous to what we observed in gene-edited *MUC2* in LS174T cells.

Even though mucin granules are the sole source of mucus in the colon, it is reasonable to assume that other goblet cell-secreted proteins not present in mucin granules could be present in polymerized secreted mucus. A major breakthrough in understanding the biology of mucin granule proteins and their interactions was the ability to image the mucin granule proteins through STED microscopy. In spite of the small size of granules [average of approximately 1 µm (42)], STED imaging conclusively demonstrated that FCGBP was a significant component present in MUC2 mucin granules. These findings bolster our findings that MUC2 and FCGBP are biosynthesized and packaged together in mucin granules and are stored in goblet-like cells for subsequent release. The STED imaging data on newly secreted mucus strands from goblet-like cells demonstrated that while both FCGBP and MUC2 are highly abundant once secreted, they partition differently and quickly lose their once-tight colocalization. It appears that both MUC2 and FCGBP segregate to specific areas within the mucus strand and quickly lose their colocalization upon polymerization. We do not know if this was due to degradation of FCGBP or loss of immunoreactivity once polymerized. In older mucus strands, both MUC2 and FCGBP were found as aggregates with no definitive structure that remained attached or was in the process of being pinched off the cell surface. While we cannot translate these findings to what occurs in the colon, the data suggest that both MUC2 and FCGBP are intimately involved in the spatial organization of the mucus gel.

The mucin granule proteome landscape revealed several interesting findings related to MAPs. Using STRING analysis protein-protein interaction predictions, all the other MAPs (CLCA1, ZG16, and TFF3) were shown to be clustered around FCGBP and not bound to MUC2 mucin. This is interesting, as it suggests the presence and fate of the MAPs in secreted mucus are highly dependent on their interaction with FCGBP. This unexpected finding and association with MUC2, lend support that the MAPs play a major role in MUC2 barrier functions where FCGBP is at the center stage. The mucin granule proteome also revealed the absence of many proteins that were found in the proteome of secreted mucus (15, 51), that could be from goblet cells or proteins derived from other mucosal epithelial cells that are embedded in the mucus layer. Given the importance of MUC2 and FCGBP that form a cluster with MAPs, we were interested in how these proteins are partitioned in secreted mucus. Using gel filtration chromatography, native secreted mucus showed the presence of MUC2 and FCGBP in the V_0_ and in all the lower molecular weight fractions under reducing conditions using Western blotting (**Figure 5B**). This suggests that upon secretion FCGBP were not tightly bound to MUC2 or were degraded. The FCGBP that remained bound to MUC2 separated in the stacking gel under reducing and non-reducing conditions. Under reducing conditions, FCGBP separated from MUC2 as two major protein bands at 70 KDa and 26 KDa. The presence of multiple FCGBP bands in the Western blots under reducing conditions is consistent with the presence of multiple autocatalytically cleaved products as shown recently (16). In addition, under reducing and non-reducing conditions, CLCA1 was abundantly expressed and associated with FCGBP and MUC2. Using CsCl density gradient ultracentrifugation, FCGBP and MUC2 were shown to be non-covalently bound in native secreted MUC2 mucus. This raises the interesting question, how does FCGBP interact with MUC2 in secreted mucus? As both molecules contain N-linked glycans, we theorize that N-linked glycan-glycan binding forms interchain bonds to stabilize the proteins. In support of this, the N-linked glycosylation inhibitor (tunicamycin) was shown to disrupt FCGBP and MUC2 colocalization and the proteins were dispersed throughout the cell cytoplasm. Moreover, on agarose and SDS-PAGE gels, there was a shift in the migration pattern of the apoproteins with a significant decrease in the total glycosylated MUC2.

To gain insights into the functional role of FCGBP and its interaction with MUC2, we followed the fate of both proteins in wound healing. Previous studies have shown structural weakening of the mucus barrier associated with loss of MUC2 and FCGBP protein prior to the onset of inflammation in UC (67). To date, no studies have looked at the protein expression of MUC2/FCGBP in colonic cells during wound healing. In wounded WT goblet-like cells, both MUC2 and FCGBP were highly colocalized to the margin of the wound with decreased mRNA expression during injury and restitution. In marked contrast, in wounded *MUC2 KO* cells, FCGBP was highly expressed and diffusely distributed throughout the cytoplasm of the cells and did not polarize to the wound margin that healed within 2 days. These cells also secreted high levels of growth factor EGF associated with increased cell migration and proliferation, supporting the proteome data that predicted stabilization and expansion of the E-cadherin adherens junction in *MUC2 KO* cells. Based on these findings, it is tempting to ascribe a functional role to cytoplasmic FCGBP in wound healing. In DSS colitis in *Muc2^+/+^* during the acute and restitutive phases of DSS colitis, even though Muc2 mRNA expression was low, Fcgbp mRNA was high with increased protein expression on day 15 as compared to *Muc2^-/-^* littermates and was associated with restitution. In *Muc2^-/-^*, Fcgbp mRNA expression was below basal levels during acute colitis and in restitution that healed slower than the *Muc2^+/+^* littermates. One plausible explanation for this is that microbiota in close contact with the surface epithelium in the absence of a mucus protective barrier in *Muc2^-/-^* littermates continually drive colitis-inhibiting Fcgbp expression (26).

As the interaction between and functional roles of MAPs and MUC2 are not well known, the findings of our study focusing on FCGBP made several important observations that have implications for innate immunity and host defense. We showed that goblet-like cells have two pools of FCGBP: one pool bound to MUC2 in mucin granules and destined to form the mucus layer and another pool free in the cytoplasm that may have distinct endogenous functions, perhaps in wound healing. Intriguingly, we revealed by using biochemical and proteomic analysis that FCGBP and MUC2 are coordinately biosynthesized, non-covalently bound, packaged within MUC2 mucin granules, and are secreted together to form part of the structural component of the mucus layer. Importantly, FCGBP revealed multiple protein-protein interactions with other MAPs including CLCA1, ZG16, and TFF3, which in turn, interacted non-covalently with MUC2 via N-linked glycans to form interchain bonds. This tenuous interaction with MUC2 perhaps made FCGBP unstable and highly susceptible to degradation in secreted mucus. The disappearance of FCGBP and MUC2 during the restitutive phase of DSS colitis suggests that both molecules are tightly regulated and may have a long-lasting impact during tissue healing and in epithelial barrier function.

## Data availability statement

The original contributions presented in the study are included in the article/**Supplementary Material**. Further inquiries can be directed to the corresponding author.

## Ethics statement

The animal study was reviewed and approved by The Health Sciences Animal Care Committee from the University of Calgary, have examined the animal care and treatment protocol (AC18-0218) and approved the experimental procedures proposed and certifies with the applicant that the care and treatment of animals used was in accordance with the principles outlined in the most recent policies on the “Guide to the Care and Use of Experimental Animals” by The Canadian Council on Animal Care.

## Author contributions

HG and KC constructed the experimental design. HG, KC, FM, and AD contributed reagents and methodical support. HG and FM performed the majority of experiments and data analysis. FM contributed to the mouse DSS colitis study. HG, AD, and KC wrote the manuscript. All authors contributed to the article and approved the submitted version.

## Supplementary material

Supplementary Figure 1 MUC2 and FCGBP protein expression are coordinately regulated *in vitro*. **(A)** Quantification of temporal MUC2 and FCGBP protein expression in LS174T goblet-like cells in culture. Protein was quantified every 2 days. *p<0.05, **p<0.01, ***p<0.001, ****p<0.0001 (n=4-8). **(B)** Quantification of LS174T goblet cell protein secretion under basal conditions and in response to the mucus secretagogue, PMA. ****p<0.0001 (n=10-12).

Supplementary Figure 2 Treatment of LS174T goblet cell with 5 μg of the N-linked glycosylation inhibitor tunicamycin dissociates FCGBP binding with MUC2. **(A)** Immunofluorescence confocal microscopy images showing MUC2 (red), FCGBP (green), and DAPI (blue). Images are representative of 3 different experiments. Scale bar = 10 μm. **(B)** Quantification of colocalization between MUC2 and FCGBP confocal microscopy images. ****p<0.0001 (n=15). **(C)** Treatment of LS174T cells with tunicamycin caused a shift in MUC2 apoprotein and FCGBP migration pattern on a 1% agarose gel or SDS-PAGE/Western blot.

Supplementary Figure 3 Proteomic analysis of purified human mucin granules from LS174T cells under non-reducing conditions. **(A)** Metascape analysis showing gene ontology enrichment of pathways utilized by each fraction under non-reducing conditions. Pathways of interest are highlighted in red. **(B)** Metascape analysis showing gene ontology biological processes associated with the proteins present in each of the 4 fractions under non-reducing conditions. Pathways of interest are highlighted in red. **(C)** Metascape analysis showing transcription factors associated with protein hits from each mucin granule fraction under non-reducing conditions. Transcription factors of interest are highlighted in red. **(D)** STRING analysis showing known protein-protein interactions for mucus-associated proteins AGR2, CLCA1, ZG16, and KLK1.

Supplementary Figure 4 MUC2 and FCGBP expression in goblet-like cells during restitution. **(A)** Quantification of confocal images from . Fluorescence of FCGBP and MUC2 in control (no wound) and in wounded cells at the wound margin and outside the wound margin (stippled open boxed areas) during restitution. ****p<0.0001 (n=6 per day). **(B)** Wounds were generated using the culture insert method. At 0 to 7 days post-inoculation, *MATH1*, *SPDEF*, and *ATF3* mRNA were analyzed by RT-PCR. GAPDH was used as a housekeeping gene. ***p<0.001, ****p<0.0001 (n=6 per day). **(C)** MUC2 FCGBP mRNA expression in healthy controls and UC colonic biopsy samples analyzed through RT-PCR. GAPDH was used as a housekeeping gene. Ctrl = healthy control; UC = active ulcerative colitis; UC-R = ulcerative colitis in remission (n=5-6 per group).

Supplementary Table 1 Shotgun proteomics proteins from WT and *MUC2 KO* LS174T cells.

Supplementary Table 2 Shotgun proteomics proteins from LS174T goblet cell granules under reducing and non-reducing conditions.

## References

[1] Cornick S Tawiah A Chadee K. Roles and regulation of the mucus barrier in the gut. Tissue Barriers (2015) 3:e982426. doi: 10.4161/21688370.2014.982426 25838985 PMC4372027

[2] McCracken VJ Lorenz RG. The gastrointestinal ecosystem: a precarious alliance among epithelium, immunity and microbiota: microreview. Cell Microbiol (2001) 3:1–11. doi: 10.1046/j.1462-5822.2001.00090.x 11207615

[3] Arike L Hansson GC. The densely O-glycosylated MUC2 mucin protects the intestine and provides food for the commensal bacteria. J Mol Biol (2016) 428:3221–9. doi: 10.1016/j.jmb.2016.02.010 PMC4982847 26880333

[4] Kim YS Ho SB. Intestinal goblet cells and mucins in health and disease: recent insights and progress. Curr Gastroenterol Rep (2010) 12:319–30. doi: 10.1007/s11894-010-0131-2 PMC2933006 20703838

[5] Johansson ME Hansson GC. Immunological aspects of intestinal mucus and mucins. Nat Rev Immunol (2016) 16:639–49. doi: 10.1038/nri.2016.88 PMC6435297 27498766

[6] Turner JR. Intestinal mucosal barrier function in health and disease. Nat Rev Immunol (2009) 9:799–809. doi: 10.1038/nri2653 19855405

[7] Johansson ME Larsson JM Hansson GC. The two mucus layers of colon are organized by the MUC2 mucin, whereas the outer layer is a legislator of host–microbial interactions. Proc Natl Acad Sci (2011) 108:4659–65. doi: 10.1073/pnas.1006451107 PMC3063600 20615996

[8] Dharmani P Srivastava V Kissoon-Singh V Chadee K. Role of intestinal mucins in innate host defense mechanisms against pathogens. J Innate Immun (2009) 1:123–35. doi: 10.1159/000163037 PMC7312850 20375571

[9] Moncada D Chadee K. Production, structure and function of gastrointestinal mucins. In: Blaser MJ Ravdin JI Greenburg HB Guerrant RL, editors. Infections of the gastrointestinal tract. Philadelphia: Lippincott Williams & Wilkins (2002). p. 57–79.

[10] Godl K Johansson MEV Lidell ME Mörgelin M Karlsson H Olson FJ. The n terminus of the MUC2 mucin forms trimers that are held together within a trypsin-resistant core fragment. J Biol Chem (2002) 277:47248–56. doi: 10.1074/jbc.M208483200 12374796

[11] Rodríguez-Piñeiro AM Bergström JH Ermund A Gustafsson JK Schütte A Johansson ME. Studies of mucus in mouse stomach, small intestine, and colon. II. gastrointestinal mucus proteome reveals Muc2 and Muc5ac accompanied by a set of core proteins. Am J Physiol Liver Physiol (2013) 305:G348–56. doi: 10.1152/ajpgi.00047.2013 PMC3761249 23832517

[12] Harada N Iijima S Kobayashi K Yoshida T Brown WR Hibi T. Human IgGFc binding protein (FcγBP) in colonic epithelial cells exhibits mucin-like structure. J Biol Chem (1997) 272:15232–41. doi: 10.1074/jbc.272.24.15232 9182547

[13] Lidell ME Hansson GC. Cleavage in the GDPH sequence of the c-terminal cysteine-rich part of the human MUC5AC mucin. Biochem J (2006) 399:121–9. doi: 10.1042/BJ20060443 PMC1570170 16787389

[14] Lidell ME Johansson ME Hansson GC. An autocatalytic cleavage in the c terminus of the human MUC2 mucin occurs at the low pH of the late secretory pathway. J Biol Chem (2003) 278:13944–51. doi: 10.1074/jbc.M210069200 12582180

[15] Johansson ME Thomsson KA Hansson GC. Proteomic analyses of the two mucus layers of the colon barrier reveal that their main component, the Muc2 mucin, is strongly bound to the fcgbp protein. J Proteome Res (2009) 8:3549–57. doi: 10.1021/pr9002504 19432394

[16] Ehrencrona E van der Post S Gallego P Recktenwald CV Rodriguez-Pineiro AM Garcia-Bonete MJ. The IgG fc-binding protein FCGBP is secreted with all GDPH sequences cleaved, but maintained by inter-fragment disulfide bonds. J Biol Chem (2021) 100871. doi: 10.1016/j.jbc.2021.100871 PMC8267560 34126068

[17] Viennois E Chen F Laroui H Baker MT Merlin D. Dextran sodium sulfate inhibits the activities of both polymerase and reverse transcriptase: lithium chloride purification, a rapid and efficient technique to purify RNA. BMC Res Notes (2013) 6:360. doi: 10.1186/1756-0500-6-360 24010775 PMC3847706

[18] Belley A Keller K Grove J Chadee K. Interaction of LS174T human colon cancer cell mucins with *Entamoeba histolytica*: an *in vitro* model for colonic disease. Gastroenterology (1996) 111:1484–92. doi: 10.1016/S0016-5085(96)70009-4 8942726

[19] Begum S Moreau F Dufour A Chadee K. *Entamoeba histolytica* exploits the autophagy pathway in macrophages to trigger inflammation in disease pathogenesis. Mucosal Immunol (2021) 14(5):1–17. doi: 10.1038/s41385-021-00408-4 33963264

[20] Zhou Y Zhou B Pache L Chang M Khodabakhshi AH Tanaseichuk O. Metascape provides a biologist-oriented resource for the analysis of systems-level datasets. Nat Commun (2019) 10:1–10. doi: 10.1038/s41467-019-09234-6 30944313 PMC6447622

[21] Szklarczyk D Gable AL Lyon D. STRING v11: protein-protein association networks with increased all rights reserved. no reuse allowed without permission. coverage, supporting functional discovery in genome-wide experimental datasets. Nucleic Acids Res (2019) 47:D607–13. doi: 10.1093/nar/gky1131 PMC6323986 30476243

[22] Moncada D Keller K Chadee K. *Entamoeba histolytica* cysteine proteinases disrupt the polymeric structure of colonic mucin and alter its protective function. Infect Immun (2003) 71:838–44. doi: 10.1128/IAI.71.2.838-844.2003 PMC145371 12540564

[23] Tiwari S Begum S Moreau F Gorman H Chadee K. Autophagy is required during high MUC2 mucin biosynthesis in colonic goblet cells to contend metabolic stress. Am J Physiol Liver Physiol (2021) 321:G489–99. doi: 10.1152/ajpgi.00221.2021 34494458

[24] Cornick S Kumar M Moreau F Gaisano H Chadee K. VAMP8-mediated MUC2 mucin exocytosis from colonic goblet cells maintains innate intestinal homeostasis. Nat Commun (2019) 10:1–14. doi: 10.1038/s41467-019-11811-8 31541089 PMC6754373

[25] Leon-Coria A Kumar M Moreau F Chadee K. Defining cooperative roles for colonic microbiota and Muc2 mucin in mediating innate host defense against entamoeba histolytica. PloS Pathog (2018) 14:e1007466. doi: 10.1371/journal.ppat.1007466 30500860 PMC6268003

[26] Leon-Coria A Kumar M Workentine M Moreau F Surette M Chadee K. Muc2 mucin and nonmucin microbiota confer distinct innate host defense in disease susceptibility and colonic injury. Cell Mol Gastroenterol Hepatol (2021) 11:77–98. doi: 10.1016/j.jcmgh.2020.07.003 32659381 PMC7596264

[27] Bu X-D Li N Tian X-Q Huang P-L. Caco-2 and LS174T cell lines provide different models for studying mucin expression in colon cancer. Tissue Cell (2011) 43:201–6. doi: 10.1016/j.tice.2011.03.002 21470648

[28] Dray-Charier N Paul A Combettes L Bouin M Mergey M Balladur P. Regulation of mucin secretion in human gallbladder epithelial cells: predominant role of calcium and protein kinase c. Gastroenterology (1997) 112:978–90. doi: 10.1053/gast.1997.v112.pm9041261 9041261

[29] Lee HW Ahn DH Crawley SC Li JD Gum JR Basbaum CB. Phorbol 12-myristate 13-acetate up-regulates the transcription of MUC2 intestinal mucin via ras, ERK, and NF-κB. J Biol Chem (2002) 277:32624–31. doi: 10.1074/jbc.M200353200 12077118

[30] Yin X Farin HF Van Es JH Clevers H Langer R Karp JM. Niche-independent high-purity cultures of Lgr5+ intestinal stem cells and their progeny. Nat Methods (2014) 11:106–12. doi: 10.1038/nmeth.2737 PMC3951815 24292484

[31] Cramer JM Thompson T Geskin A LaFramboise W Lagasse E. Distinct human stem cell populations in small and large intestine. PloS One (2015) 10:e0118792. doi: 10.1371/journal.pone.0118792 25751518 PMC4353627

[32] Van der Sluis M De Koning BAE De Bruijn ACJM Velcich A Meijerink JPP Van Goudoever JB. Muc2-deficient mice spontaneously develop colitis, indicating that MUC2 is critical for colonic protection. Gastroenterology (2006) 131:117–29. doi: 10.1053/j.gastro.2006.04.020 16831596

[33] Tawiah A Moreau F Kumar M Tiwari S Falguera J Chadee K. High MUC2 mucin biosynthesis in goblet cells impedes restitution and wound healing by elevating endoplasmic reticulum stress and altered production of growth factors. Am J Pathol (2018) 188:2025–41. doi: 10.1016/j.ajpath.2018.05.013 29935164

[34] Allaire JM Morampudi V Crowley SM Stahl M Yu H Bhullar K. Frontline defenders: goblet cell mediators dictate host-microbe interactions in the intestinal tract during health and disease. Am J Physiol Liver Physiol (2018) 314:G360–77. doi: 10.1152/ajpgi.00181.2017 PMC5899238 29122749

[35] Birchenough G Schroeder BO Bäckhed F Hansson GC. Dietary destabilisation of the balance between the microbiota and the colonic mucus barrier. Gut Microbes (2019) 10:246–50. doi: 10.1080/19490976.2018.1513765 PMC6546334 30252606

[36] Jakobsson HE Rodríguez-Piñeiro AM Schütte A Ermund A Boysen P Bemark M. The composition of the gut microbiota shapes the colon mucus barrier. EMBO Rep (2015) 16:164–77. doi: 10.15252/embr.201439263 PMC4328744 25525071

[37] Reikvam DH Erofeev A Sandvik A Grcic V Jahnsen FL Gaustad P. Depletion of murine intestinal microbiota: effects on gut mucosa and epithelial gene expression. PloS One (2011) 6:e17996. doi: 10.1371/journal.pone.0017996 21445311 PMC3061881

[38] Wlodarska M Willing B Keeney KM Menendez A Bergstrom KS Gill N. Antibiotic treatment alters the colonic mucus layer and predisposes the host to exacerbated *Citrobacter rodentium*-induced colitis. Infect Immun (2011) 79:1536–45. doi: 10.1128/IAI.01104-10 PMC3067531 21321077

[39] Arike L Holmén-Larsson J Hansson GC. Intestinal Muc2 mucin O-glycosylation is affected by microbiota and regulated by differential expression of glycosyltranferases. Glycobiology (2017) 27:318–28. doi: 10.1093/glycob/cww134 PMC5444243 28122822

[40] Bergström A Kristensen MB Bahl MI Metzdorff SB Fink LN Frøkiær H. Nature of bacterial colonization influences transcription of mucin genes in mice during the first week of life. BMC Res Notes (2012) 5:1–7. doi: 10.1186/1756-0500-5-402 22857743 PMC3465226

[41] Johansson ME Jakobsson HE Holmén-Larsson J Schütte A Ermund A Rodríguez-Piñeiro AM. Normalization of host intestinal mucus layers requires long-term microbial colonization. Cell Host Microbe (2015) 18:582–92. doi: 10.1016/j.chom.2015.10.007 PMC4648652 26526499

[42] Cornick S Moreau F Chadee K. *Entamoeba histolytica* cysteine proteinase 5 evokes mucin exocytosis from colonic goblet cells via αvβ3 integrin. PloS Pathog (2016) 12:e1005579. doi: 10.1371/journal.ppat.1005579 27073869 PMC4830554

[43] Vicidomini G Bianchini P Diaspro A. STED super-resolved microscopy. Nat Methods (2018) 15:173–82. doi: 10.1038/nmeth.4593 29377014

[44] Oliveros JC. VENNY. an interactive tool for comparing lists with Venn diagrams (2007). Available at: http://bioinfogp.cnb.csic.es/tools/venny/index.html.

[45] Cornick S Moreau F Gaisano HY Chadee K. *Entamoeba histolytica*-induced mucin exocytosis is mediated by VAMP8 and is critical in mucosal innate host defense. MBio (2017) 8:e01323–17. doi: 10.1128/mBio.01323-17 PMC5626970 28974617

[46] Perrais M Pigny P Copin M-C Aubert JP Van Seuningen I. Induction of MUC2 and MUC5AC mucins by factors of the epidermal growth factor (EGF) family is mediated by EGF receptor/Ras/Raf/extracellular signal-regulated kinase cascade and Sp1. J Biol Chem (2002) 277:32258–67. doi: 10.1074/jbc.M204862200 12077147

[47] Gul IS Hulpiau P Saeys Y Van Roy F. Evolution and diversity of cadherins and catenins. Exp Cell Res (2017) 358:3–9. doi: 10.1016/j.yexcr.2017.03.001 28268172

[48] Ten Hagen KG Fritz TA Tabak LA. All in the family: the UDP-GalNAc: polypeptide n-acetylgalactosaminyltransferases. Glycobiology (2003) 13:1R–16R. doi: 10.1093/glycob/cwg007 12634319

[49] Nagae M Kizuka Y Mihara E Kitago Y Hanashima S Ito Y. Structure and mechanism of cancer-associated n-acetylglucosaminyltransferase-V. Nat Commun (2018) 9:1–12. doi: 10.1038/s41467-018-05931-w 30140003 PMC6107550

[50] Bergstrom KSB Xia L. Mucin-type O-glycans and their roles in intestinal homeostasis. Glycobiology (2013) 23:1026–37. doi: 10.1093/glycob/cwt045 PMC3858029 23752712

[51] van der Post S Jabbar KS Birchenough G Arike L Akhtar N Sjovall H. Structural weakening of the colonic mucus barrier is an early event in ulcerative colitis pathogenesis. Gut (2019) 68:2142–51. doi: 10.1136/gutjnl-2018-317571 PMC6872445 30914450

[52] Chadee K Petri WA Innes DJ Ravdin JI. Rat and human colonic mucins bind to and inhibit adherence lectin of entamoeba histolytica. J Clin Invest (1987) 80:1245–54. doi: 10.1172/JCI113199 PMC442377 2890655

[53] Carlstedt I Lindgren H Sheehan JK Ulmsten U Wingerup L. Isolation and characterization of human cervical-mucus glycoproteins. Biochem J (1983) 211:13–22. doi: 10.1042/bj2110013 6409086 PMC1154324

[54] Thornton DJ Khan N Sheehan JK. Separation and identification of mucins and their glycoforms. Glycoprotein Methods Protoc (2000) 125:77–85. doi: 10.1385/1-59259-048-9:077 10820751

[55] Press AG Hauptmann IA Hauptmann L Fuchs B Fuchs M Ewe K. Gastrointestinal pH profiles in patients with inflammatory bowel disease. Aliment Pharmacol Ther (1998) 12:673–8. doi: 10.1046/j.1365-2036.1998.00358.x 9701532

[56] Marteyn B Scorza FB Sansonetti PJ Tang C. Breathing life into pathogens: the influence of oxygen on bacterial virulence and host responses in the gastrointestinal tract. Cell Microbiol (2011) 13:171–6. doi: 10.1111/j.1462-5822.2010.01549.x 21166974

[57] Huang C Jacobson K Schaller MD. A role for JNK-paxillin signaling in cell migration. Cell Cycle (2004) 3:3–5. doi: 10.4161/cc.3.1.601 14657652

[58] Angelozzi M de Charleroy CR Lefebvre V. EdU-based assay of cell proliferation and stem cell quiescence in skeletal tissue sections. Skelet Dev Repair Methods Protoc (2021), 357–65. doi: 10.1007/978-1-0716-1028-2_21 PMC11783623 33197025

[59] Tremel A Cai A Tirtaatmadja N Hughes BD Stevens GW Landman KA. Cell migration and proliferation during monolayer formation and wound healing. Chem Eng Sci (2009) 64:247–53. doi: 10.1016/j.ces.2008.10.008

[60] Kumar M Kissoon-Singh V Leon-Coria A Moreau F Chadee K. The probiotic mixture VSL# 3 reduces colonic inflammation and improves intestinal barrier function in Muc2 mucin deficient mice. Am J Physiol Circ Physiol (2017) 312:G34–45. doi: 10.1152/ajpgi.00298.2016 27856417

[61] Dharmani P Leung P Chadee K. Tumor necrosis factor-α and Muc2 mucin play major roles in disease onset and progression in dextran sodium sulphate-induced colitis. PloS One (2011) 6:e25058. doi: 10.1371/journal.pone.0025058 21949848 PMC3176316

[62] Tawiah A Cornick S Moreau F Gorman H Kumar M Tiwari S. High MUC2 mucin expression and misfolding induce cellular stress, reactive oxygen production, and apoptosis in goblet cells. Am J Pathol (2018) 188:1354–73. doi: 10.1016/j.ajpath.2018.02.007 29545196

[63] Rodríguez-Piñeiro AM van der Post S Johansson ME Thomsson KA Nesvizhskii AI Hansson GC. Proteomic study of the mucin granulae in an intestinal goblet cell model. J Proteome Res (2012) 11:1879–90. doi: 10.1021/pr2010988 PMC3292267 22248381

[64] Keller K Olivier M Chadee K. The fast release of mucin secretion from human colonic cells induced by *Entamoeba histolytica* is dependent on contact and protein kinase c activation. Arch Med Res (1992) 23:217–21. 1340298

[65] Van Seuningen I Pigny P Perrais M Porchet N Aubert JP. Transcriptional regulation of the 11p15 mucin genes. towards new biological tools in human therapy, in inflammatory diseases and cancer. Front Biosci (2001) 6:D1216–34. 10.2741/seuning 11578973

[66] Stelzer G Rosen N Plaschkes I Zimmerman S Twik M Fishilevich S. The GeneCards suite: from gene data mining to disease genome sequence analyses. Curr Protoc Bioinforma (2016) 54:1–30. doi: 10.1002/cpbi.5 27322403

[67] Johansson ME Gustafsson JK Holmén-Larsson J Jabbar KS Xia L Xu H. Bacteria penetrate the normally impenetrable inner colon mucus layer in both murine colitis models and patients with ulcerative colitis. Gut (2014) 63:281–91. doi: 10.1136/gutjnl-2012-303207 PMC3740207 23426893

