# Supplementary figures and images for: IgGFc-binding protein and MUC2 mucin produced by colonic goblet-like cells spatially interact non-covalently and regulate wound healing
Source: Front Immunol. 2023 Jun 8;14:1211336. doi: 10.3389/fimmu.2023.1211336 (PMC10285406; doi:10.3389/fimmu.2023.1211336)

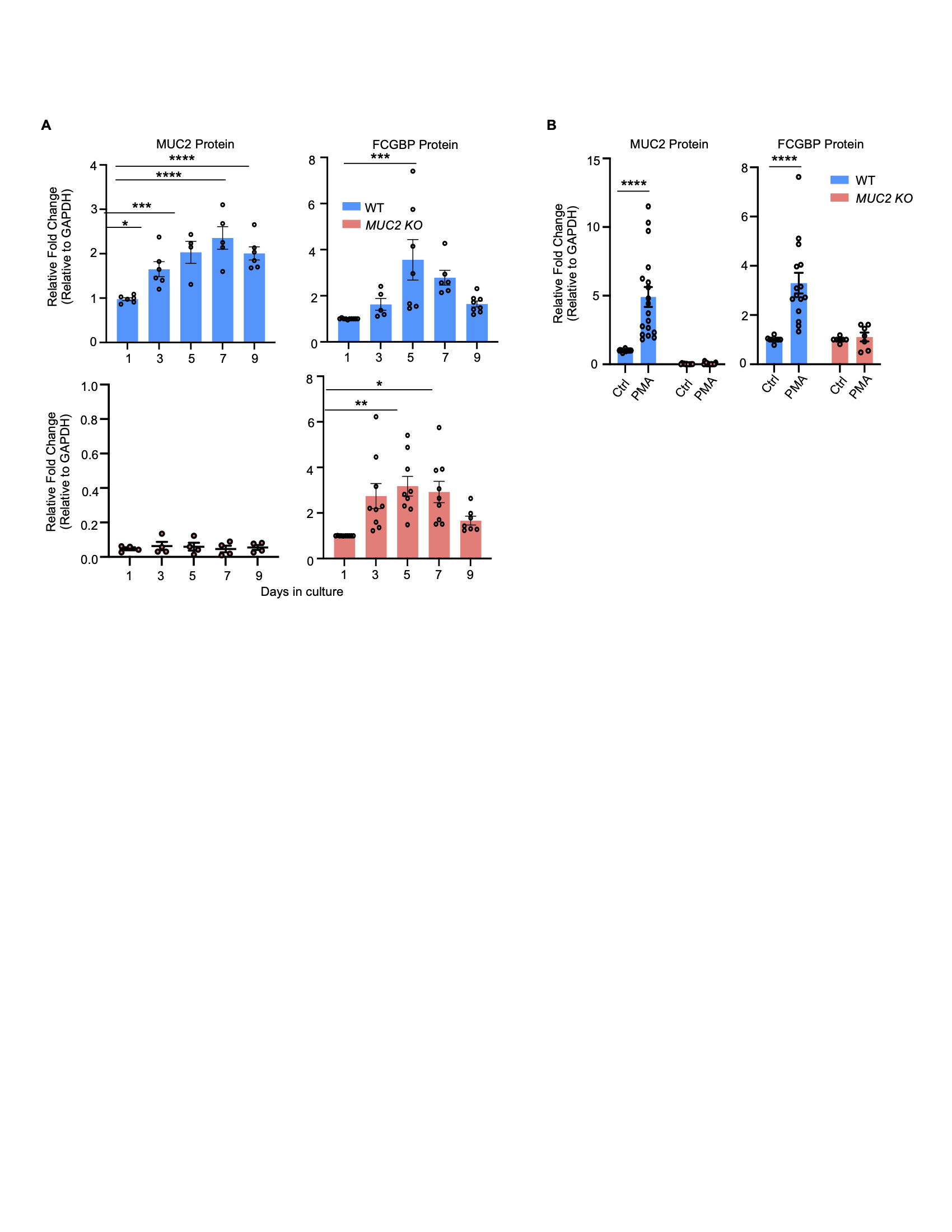

Supplement: Supplementary Figure 1 — MUC2 and FCGBP protein expression are coordinately regulated in vitro. (A) Quantification of temporal MUC2 and FCGBP protein expression in LS174T goblet-like cells in culture. Protein was quantified every 2 days. *p<0.05, **p<0.01, ***p<0.001, ****p<0.0001 (n=4-8). (B) Quantification of LS174T goblet cell protein secretion under basal conditions and in response to the mucus secretagogue, PMA. ****p<0.0001 (n=10-12). [file Image_1.tiff]
